# A Web-Based Intervention for Reducing Sexually Transmitted Infections and Substance Use During Pregnancy: Randomized Controlled Trial

**DOI:** 10.2196/95944

**Published:** 2026-07-08

**Authors:** Golfo Tzilos Wernette, Ananda Sen, Dongru Chen, Kristina Countryman, Okeoma Mmeje, Quyen M Ngo, Katherine J Gold, Christopher W Kahler, Caron Zlotnick

**Affiliations:** 1 Department of Family Medicine Medical School University of Michigan Ann Arbor, MI United States; 2 Department of Biostatistics School of Public Health University of Michigan Ann Arbor, MI United States; 3 Department of Obstetrics and Gynecology Medical School University of Michigan Ann Arbor, MI United States; 4 Health Behavior and Health Equity School of Public Health University of Michigan Ann Arbor, MI United States; 5 Butler Center for Research Hazelden Betty Ford Foundation Center City, MN United States; 6 Department of Psychiatry and Human Behavior Alpert Medical School of Brown University Providence, RI United States; 7 Department of Behavioral and Social Sciences Center for Alcohol and Addiction Studies Brown University Providence, RI United States; 8 Department of Medicine Women and Infants Hospital Providence, RI United States; 9 Department of Psychiatry and Mental Health University of Cape Town Cape Town South Africa

**Keywords:** alcohol use, drug use, cannabis use, sexually transmitted infections, technology-delivered, pregnancy

## Abstract

**Background:**

Sexually transmitted infections (STIs) are at a record high in the United States and are a significant health problem for childbearing women. Rates of substance use, particularly cannabis and opioid use, have increased in recent years and are linked to negative health consequences for pregnant women and their infants. Addressing these health concerns together during this vulnerable time is a priority.

**Objective:**

This study aims to test whether the Health Check-Up for Expectant Moms (HCEM), an innovative, theory-driven, technology-delivered, and fully automated brief intervention, reduced condomless sex and STI risk, alcohol, or drug use, compared to a control condition in pregnant women seeking prenatal care.

**Methods:**

We recruited a sample of 176 pregnant women (all were at risk for alcohol or drug use or STIs during pregnancy) from clinics and using social media campaigns (Facebook and Instagram) in the state of Michigan and randomized them to the motivational interviewing-consistent HCEM intervention or to an attention-, time-, and information-matched control condition delivered using the same technology platform. We followed these women at 2 and 6 months after the initial intervention visit. Primary outcomes included self-report assessment of alcohol, drug, or cannabis use and unprotected sexual occasions during pregnancy.

**Results:**

A total of 88 women were randomized to the intervention, and 88 to the control condition. Cannabis use was the most prevalent substance reported during pregnancy; a total of 35.2% (62/176) reported recent use (within the last 90 days) at baseline, with 10.2% (18/176) reporting use in the month prior to baseline. There were significant reductions in alcohol and cannabis use over time during pregnancy (at 2 and 6 months compared to baseline) in both HCEM and control groups; however, these reductions were not significantly different between conditions (time-by-arm interaction), and most were sustained from spontaneous reductions reported in the month before study enrollment. Moreover, there were no statistically significant differences in the change pattern of condomless sex across the groups at either follow-up.

**Conclusions:**

There are many potential benefits of a technology-delivered approach to support the behavioral health of pregnant women in a private and convenient way. Our sample was largely low-risk, and as such, an intervention effect may have been impossible to observe given substantial self-change. Future trials are needed to examine efficacy in other samples of pregnant women with a higher risk of current alcohol or drug use.

**Trial Registration:**

ClinicalTrials.gov NCT03826342; https://clinicaltrials.gov/study/NCT03826342

**International Registered Report Identifier (IRRID):**

RR2-10.2196/30367

## Introduction

Substance use during pregnancy is associated with several negative consequences for both the pregnant woman and the developing fetus, including increasing the incidence of sexually transmitted infections (STIs) [1-3], postpartum depression [4-6], and significant fetal and neonatal complications [7-9]. Among pregnant women who use substances, the co-use of other substances is common [10]; 38% of pregnant women who report current alcohol use also report use of one or more other substances (mostly tobacco and cannabis) [7]. Cannabis is the most frequently used drug during pregnancy [11,12], and several recent meta-analyses show that newborns with exposure to cannabis in utero were more likely to be born preterm, have a low birth weight, and require neonatal intensive care compared to newborns without exposure [13-17]. Alcohol use during pregnancy can lead to life-long adverse effects for the infant, including fetal alcohol spectrum disorders [18]. There has been a dramatic 5-fold national increase in opioid use during pregnancy [19], leading to a surge in the incidence of neonatal abstinence syndrome [20]. This postnatal drug withdrawal syndrome includes central nervous system irritability [21].

STIs remain at record highs in the United States [22], and STI risk continues to be a critical and costly health problem for women [23], especially for pregnant women who can transmit infections to their babies [24]. STIs can negatively impact both the woman and baby and can result in premature birth, low birthweight, and fetal death [25,26]. A pregnant woman is at risk of transmitting certain STIs (like HIV) to her infant during pregnancy, delivery, or breastfeeding, especially if untreated [27]. Importantly, substance use and risky sexual behavior, together, contribute significantly to the acquisition of STIs, and this is especially concerning in vulnerable populations including during pregnancy [3,28].

Because the majority (97%) of pregnant women receive at least some prenatal care [29,30], the Centers for Disease Control and Prevention (CDC) and the American College of Obstetricians (ACOG) have identified the perinatal period as an urgent time for STI screening, prevention, and subsequent behavior change, including substance use [31,32]. Due to increased use nationwide, the ACOG recently issued a strong recommendation specifically for universal cannabis use screening during pregnancy through self-report rather than drug testing to prevent racial disparities (as significant racial disparities in drug testing are well documented) [33]. Further, postpartum women are vulnerable to increased risk of STIs associated with high-risk sexual behaviors, including inconsistent condom use [34], alcohol use (with national data suggesting prevalence rates as high as 30-49%), and drug use (with prevalence rates in the range of 4-9%) [35]. To optimize postpartum care, ACOG has recommended proactively addressing these risky health behaviors during pregnancy, given that as many as 40% of women in the United States do not receive postpartum care [36]. Intervening on health concerns during a natural life event, such as pregnancy, has been shown to be an ideal time to introduce behavior change, as women may be more motivated to adopt healthier behaviors for the well-being of the child [37,38].

To this end, technology-delivered screening and brief intervention (SBI) is a confidential, self-directed, low-cost intervention that has several notable strengths for working with women in the perinatal period. The use of computer-assisted SBI for the disclosure of STI risk has been empirically supported in terms of encouraging the disclosure of sexual risk-taking and additional STI testing [39]. This is of particular importance because web-based interventions have the potential to overcome known barriers to seeking care among pregnant women, such as fear of disclosure and stigma [40-42]. Furthermore, a recent review suggests that the use of electronic screening, brief intervention, or referral to treatment for substance use may be an effective and more equitable approach for overcoming traditional barriers to care that prevent women with these risk factors from receiving treatment during pregnancy [43].

The Health Check-Up for Expectant Moms (HCEM) is an innovative, theory-driven, technology-delivered brief intervention—a 3-interaction intervention consisting of a single session plus two booster sessions—designed to reduce alcohol or drug use and risk for STIs during pregnancy. In the HCEM pilot randomized controlled trial, we found very high ratings of acceptability in our sample of 50 pregnant women. We noted that the HCEM was associated with a significantly larger reduction in any cannabis or alcohol use during pregnancy compared with participants in the control group [44]. To our knowledge, apart from our pilot work, no other studies have assessed web-based STI prevention interventions exclusively in nontreatment-seeking pregnant women at risk for substance use. Building upon this pilot work, we report the results of our randomized controlled trial testing whether the HCEM reduced condomless sex or STI risk, alcohol or drug use, more than a control condition in a larger, more diverse sample of pregnant women seeking prenatal care.

## Methods

### Participant Recruitment and Characteristics

Initial participant recruitment took place in-person at family medicine and obstetrician & gynecology clinics between April 2019 and March 2020 (n=81). Due to the SARS-CoV-2 pandemic, new recruitment stopped until August 2020, to allow time to make the necessary protocol and software changes for complete remote delivery (see previous paper for more detail) [45]. Remote recruitment began in August 2020 and ended in September 2023 (n=95). Remote recruitment included calling, texting, or emailing existing clinic patients and social media advertising (Facebook and Instagram) throughout the state of Michigan. Study details at recruitment were limited; the study was simply referred to as a “healthy pregnancy” study, with full details reviewed after a participant was deemed eligible. Our procedures for social media recruitment included several verification measures (eg, phone calls, IP address checks, etc.) to avoid fraudulent and/or multiple enrollment attempts [46]. Interested and verified women were sent the study screener via web-link through the university-secured and encrypted website Qualtrics, to ensure confidentiality and safety. Because participation in this study required a smartphone, computer, or device with internet access, computer and internet literacy were a “de facto” eligibility criterion. Participants were compensated for their time, whether they were eligible or not. Participants completing the study screener were given a US $5 gift card. Enrolled participants could earn up to US $220 for completing all study tasks.

### Ethical Considerations

The study was approved on April 23, 2018 (HUM00143896) by the Institutional Review Board of the University of Michigan Medical School in Ann Arbor, MI, and is registered on ClinicalTrials.gov (NCT03826342). All participants went through a detailed informed consent process with a research staff member before being enrolled in the study. Informed consent was obtained face-to-face with signatures pre-SARS-CoV-2, and over the phone with verbal consent post-SARS-CoV-2.

### Web-Based Screening and Randomization Methods

Using simple randomization, we computer-randomized 176 pregnant women who met study inclusion criteria into the HCEM intervention (n=88) or an attention, time, and information-matched control (n=88) condition. Web-based software randomization and intervention delivery were completed using the computerized intervention authoring system (CIAS), version 2.0, developed by Ondersma et al [47]. Specifically, inclusion criteria included a combination of questions assessing risk for substance use and sexual health risk behaviors, placing women at risk for STIs. Risk of substance use during pregnancy was determined using a combination of 3 substance use screeners validated for use during pregnancy including, the substance use profile-pregnancy (3 items; one or more affirmative responses indicate a positive screen in low-risk populations) [48], National Institute on Drug Abuse Quick Screen V.1/Modified-ASSIST V.2 (2 items; one or more affirmative responses indicates risk) [49], and the tolerance, annoyed, cut down, eye opener (4-items; T is 2 points and A, C, E are 1 point each. 2 points or more considered positive screen) [50]. To meet the substance use risk criteria for HCEM, women had to screen positive on at least one of these validated screeners above. Eligibility for STI risk behavior included recent (within the last 30 days) condomless vaginal sex, and at least one of the following: (1) multiple sexual partners within the last 6 months, (2) a new sexual partner since becoming pregnant, or (3) uncertainty of the current partner’s monogamy. This was a change to our original (pre-SARS-CoV-2) inclusion criteria, where STI risk behavior was measured solely by self-reported recent (within the last 30 days) condomless vaginal sex (substance use risk criteria were the same). Due to participant feedback and the overall low rate of STI risk behavior observed in the data up to that point, we reviewed empirically supported methodologies [51,52] and aligned with the CDC recommendations [53] to increase the threshold for the STI risk behavior eligibility.

### Biological Samples

We collected biological hair samples (Psychemedics, Inc) and/or urine samples in person at the baseline and 6-month follow-up assessment to corroborate self-reports of drug use (a secondary outcome). The hair samples were obtained by a trained study RA, collected from cosmetically undetectable areas on the scalp, and sent off-site for analysis. All biological sample collection was suspended during the SARS-COV-2 pandemic, and therefore, this data were incomplete for all participants.

### Procedures

Participants completed the assessment and intervention or control sessions in the clinic on the study iPad (pre-SARS-CoV-2) or on a personal smartphone, computer, or device through a web link to the study website (post-SARS-CoV-2). Booster sessions were emailed to participants to be completed independently using a web link to the study website, both pre- and post-SARS-CoV-2. Reminders by phone, text, or email to complete booster sessions were sent as needed until the 2-month follow-up visit, after which booster sessions were no longer available to complete. Whether in-person or remote, a research study team member was available (during regular business hours) to help participants troubleshoot any technical problems. All participant contact (call, text, or email) was conducted using university-secured and encrypted websites to ensure participant confidentiality and safety.

Study staff members were blinded to the arm allocations of the participants they interviewed at the 2- and 6-month follow-up visits. This meant that the staff member who conducted the baseline session for a participant could not conduct any follow-up visits for that same participant. Participants were not told which arm they were randomized to; however, it is possible for them to have inferred it after reading the informed consent document, as it detailed the 2 study conditions.

### Study Measures for Primary Outcomes

Participants completed an approximately 25-minute assessment session before starting the intervention or control session. The assessment battery was designed to minimize assessment with the time, attention, and information-matched control group to account for concerns regarding the motivational properties of assessment [54,55]. All of the self-report measures have good psychometric properties:

1. Demographic information included age, race, ethnicity, marital or partner status, parity, employment, and socioeconomic status. Web-based collection was used both pre– and post–SARS-COV-2 for all demographic information.
2. Timeline follow-back (TLFB) calendar interview was conducted by the research assistant either in-person (pre-SARS-CoV-2) or remotely by phone. Data for the TLFB was entered into a database only accessible by the research team, which differs from other assessment measures that were collected through direct input from the participant either on the study iPad or via a web link to the study website. The TLFB is a calendar-assisted structured interview that provides a way to cue memory so that accurate recall is enhanced for event-level data; it has been used to assess sexual risk-taking [56,57]. It assessed sexual health behaviors, placing women at risk for STIs at baseline and follow-up assessments. A partner-by-partner assessment of sexual behaviors and condom use was made for each of the participants’ sexual partners and partner type (casual, main). Condomless sex was assessed in the 90 days prior to the baseline, as well as for each of the subsequent assessments (at 2- and 6-month postbaseline, antenatally). The 90-day time period was selected because of research supporting reliable reporting of sexual events and the number of partners over this time period [58]. The TLFB was also used to measure drinks per drinking day, percent days abstinent, and heavy episodic drinking episodes; it was also used to measure other drug use, including cannabis use.

### Treatment Conditions

#### HCEM

The HCEM is a 3-interaction intervention, including a 40-minute session that is consistent with motivational interviewing (MI), plus two brief sessions (15-20 minutes) that are also theory-driven and derived from empirical support. MI is a client-centered counseling style that facilitates internal motivation to change through alignment of behavior change with deeply held beliefs, values, and goals. MI has been found to be effective for STI risk reduction [59-63] and is consistent with self-determination theory, as it uses evolving readiness and self-efficacy to change [64]. The focus of the intervention session is on reducing the key behavioral outcomes of unprotected sex and alcohol and drug use during pregnancy. The session provides a tutorial guided by a narrator who interacts in a collaborative, MI-consistent style. HCEM includes short video testimonials of women who had an STI, how it affected them during pregnancy, and how they sought support.

The components of the HCEM intervention and boosters are highly specific to the perinatal setting and entail increasing knowledge and targeting risk perceptions about STI transmission and prevention during pregnancy (which includes education about alcohol and drug use, for example, the health risks associated with cannabis use on the infant), increasing intrinsic motivation to act during pregnancy based on that knowledge, and ensuring sufficient behavioral skills (self-efficacy) to change behavior (eg, increase condom use and decrease substance use) during pregnancy, as self-efficacy can be a strong predictor of condom use in women [65]. In order to enhance behavioral skills and use of resources for STI risk reduction, a video demonstrates male and female condom applications with anatomical models as well as strategies to respond more assertively within a sexual context during pregnancy. All participants randomized to HCEM complete an optional personalized plan designed to increase awareness of risk factors in the woman’s life, as well as partner substance use, which may increase these risks for the woman during pregnancy. All participants are provided an up-to-date resource list for alcohol and drug use treatment providers and sexual health clinics for each county of recruitment.

#### Booster Sessions

Within 1 month of completing the baseline session, participants in both conditions complete 2, 15-20-minute booster sessions, also delivered remotely. For participants in the HCEM condition, the booster sessions review their own personalized plan and identify any challenges or barriers to increasing healthy behaviors (ie, increasing condom use, improving supports). Booster sessions explicitly target the prevention or reduction of STI risk behaviors, including alcohol and drug use during the postpartum period, when prevalence of these behaviors increases, and address health behaviors associated with postpartum substance use (eg, risks of substance use while breastfeeding).

#### Information-Matched Control Condition

The purpose of our well-validated control condition was simply to control for time, attention, and information effects and has been used successfully in previous behavioral health studies [44,66-69], including in our prior HCEM trial [44]. The content consists of a series of questions regarding television show preferences and viewing a brief series of videos of popular entertainers and shows, with subsequent requests for ratings of subjective preference. Participants randomized to the control condition also received facts about alcohol and drug use and risky sex during pregnancy through informational brochures from the National Institutes of Health and the CDC, which also provided face validity. Participants in the control condition receive booster content that is similar to the baseline content.

### Statistical Analysis

We summarized key study variables through descriptive statistics. Participant characteristics and outcomes at 3 timepoints during pregnancy were compared between control and intervention groups by means of 2-sample, 2-tailed *t* tests (continuous), Wilcoxon rank sum tests, chi-square tests, and Fisher exact tests, as appropriate. The primary behaviors of interest were alcohol or drug use (primarily cannabis use) and unprotected sexual occasions, as assessed by the TLFB.

### Sample Size

We estimated power based on our R21 study and calculated it using extensive simulation. For all the outcomes, data were simulated 1000 times using appropriate statistical models and estimates from the pilot data. Power was assessed for the effects of interest empirically by the proportion of times the corresponding coefficient was deemed statistically significant at 5% level. To illustrate, for unprotected sex, the baseline average number of days of unprotected sex was 36 based on the pilot data. Assuming that the average number of days of unprotected sex drops to 32 and 29 in the control and HCEM groups, respectively (similar to that obtained in the pilot data) at any of the follow-up points, using 100 subjects per arm, the difference in the reduction in the groups will be deemed as statistically significant with more than 90% power using a Poisson regression model. The differences for alcohol use and drug use were more prominent in our R21 data, and we anticipated being well powered for these aims using the same sample size. Similar effects were obtained when the dichotomous counterparts were simulated using a clustered logistic regression model. Assuming a 20% loss to follow-up in either arm, we plan to recruit 125 participants per arm. For all the outcomes, our proposed sample size was deemed adequate for detecting small to moderate effect sizes [70].

For each participant, we report a binary indicator of any alcohol use (yes or no), drug use (primarily cannabis use) at any time over the 90-day TLFB period at 3 time points antenatally (baseline, 2-month, and 6-month follow-up). Baseline included both pre-pregnancy and pregnancy use. Mixed logistic regressions with a generalized estimating equation approach were used to investigate differences in the change of alcohol use, drug use, and cannabis use between study arms. A mixed negative binomial regression with a generalized estimating equation approach was used to investigate differences in the change of unprotected sex frequency between study arms. All models included study arm, time, and an interaction between arm and time as covariates, with a random intercept to account for intrasubject correlation. The models were further adjusted for age and marital status, where marital status was created as a 4-category covariate (legally married, living together but not married, single or separated, or widowed or divorced) by collapsing certain sparse categories. This was not an a priori covariate; it was adjusted due to the significant difference between the 2 arms observed during analysis. Although receipt of public assistance and family income were significantly different between the intervention and control groups (Table 1), they were not included in the regression models, since they were significantly associated with marital status. Because assessment window lengths varied across participants and timepoints, we included the log of the assessment duration as a time-varying offset in the negative binomial models. Analyses were conducted according to the intent-to-treat principle.

**Table 1:** Participant characteristics.

Demographics			All (N=176)		Intervention (n=88)		Control (n=88)	*P* value^a^
Age (years), mean (SD)			30.2 (5)		30.3 (4.9)		30.1 (5.1)	.81
Years of schooling completed, n (%)								.86
	0-8 years	7 (4)		3 (3)		4 (5)		
	9-11 years	9 (5)		4 (5)		5 (6)		
	12 years	25 (14)		12 (14)		13 (15)		
	13-14 years	28 (16)		14 (16)		14 (16)		
	15-16 years	33 (19)		20 (23)		13 (15)		
	More than 16 years	74 (42)		35 (40)		39 (44)		
Education, n (%)								.51
	Didn’t graduate from high school	11 (6)		5 (6)		6 (7)		
	High school graduate or general educational development (GED)	23 (13)		9 (10)		14 (16)		
	Went to technical or trade school	8 (5)		2 (2)		6 (7)		
	Some college	33 (19)		17 (19)		16 (18)		
	College graduate	49 (28)		28 (32)		21 (24)		
	Postgraduate	52 (30)		27 (31)		25 (28)		
Employment status, n (%)								>.99
	Full time	99 (56)		50 (57)		49 (56)		
	Part time	27 (15)		14 (16)		13 (15)		
	Student	5 (3)		2 (2)		3 (3)		
	Stay-at-home mom or unemployed	45 (26)		22 (25)		23 (26)		
Adults at home, n (%)								.15
	Only myself	28 (16)		9 (10)		19 (22)		
	1 other adult and myself	134 (76)		70 (80)		64 (73)		
	2 other adults and myself	9 (5)		6 (7)		3 (3)		
	3 other adults and myself	3 (2)		2 (2)		1 (1)		
	4 other adults and myself	1 (1)		1 (1)		0 (0)		
	6 or more other adults and myself	1 (1)		0 (0)		1 (1)		
Children, n (%)								.06
	0	74 (42)		42 (48)		32 (36)		
	1	54 (31)		31 (35)		23 (26)		
	2	28 (16)		10 (11)		18 (20)		
	3	11 (6)		3 (3)		8 (9)		
	4	3 (2)		1 (1)		2 (2)		
	5	2 (1)		1 (1)		1 (1)		
	6 or more	4 (2)		0 (0)		4 (5)		
Family income, n (%)								.03
	<US $50,000	76 (43)		31 (35)		45 (51)		
	US $50,000 +	100 (57)		57 (65)		43 (49)		
Receive public assistance, n (%)								.001
	Yes	56 (32)		18 (20)		38 (43)		
	No	120 (68)		70 (80)		50 (57)		
	Pregnancy weeks, mean (SD)	13.8 (4.4)		13.3 (4.4)		14.2 (4.5)		.18
Race, n (%)								.56
	White	112 (64)		61 (69)		51 (58)		
	Native American or Native Alaskan	1 (1)		1 (1)		0		
	Black	45 (26)		18 (20)		27 (31)		
	Asian	4 (2)		2 (2)		2 (2)		
	Bi-racial	7 (4)		3 (3)		4 (5)		
	Other	7 (4)		3 (3)		4 (5)		
Ethnicity, n (%)								.07
	Latina	17 (10)		5 (6)		12 (14)		
	Non-Latina	159 (90)		83 (94)		76 (86)		
Current marital status, n (%)								.006
	Single-never married	41 (23)		20 (23)		21 (24)		
	Living together but not married	33 (19)		10 (11)		23 (26)		
	Legally married	95 (54)		57 (65)		38 (43)		
	Separated	2 (1)		1 (1)		1 (1)		
	Divorced	4 (2)		0 (0)		4 (5)		
	Widowed	1 (1)		0 (0)		1 (1)		
Recruitment methods, n (%)								.99
	In-person clinic	83 (47)		42 (48)		41 (47)		
	Remote clinic	73 (41)		36 (41)		37 (42)		
	Social media	20 (11)		10 (11)		10 (11)		

^a^Differences between the study arms using 2-tailed *t* tests, chi-square tests, or Fisher exact tests, as appropriate.

## Results

### Participant Characteristics

**Figure 1:**
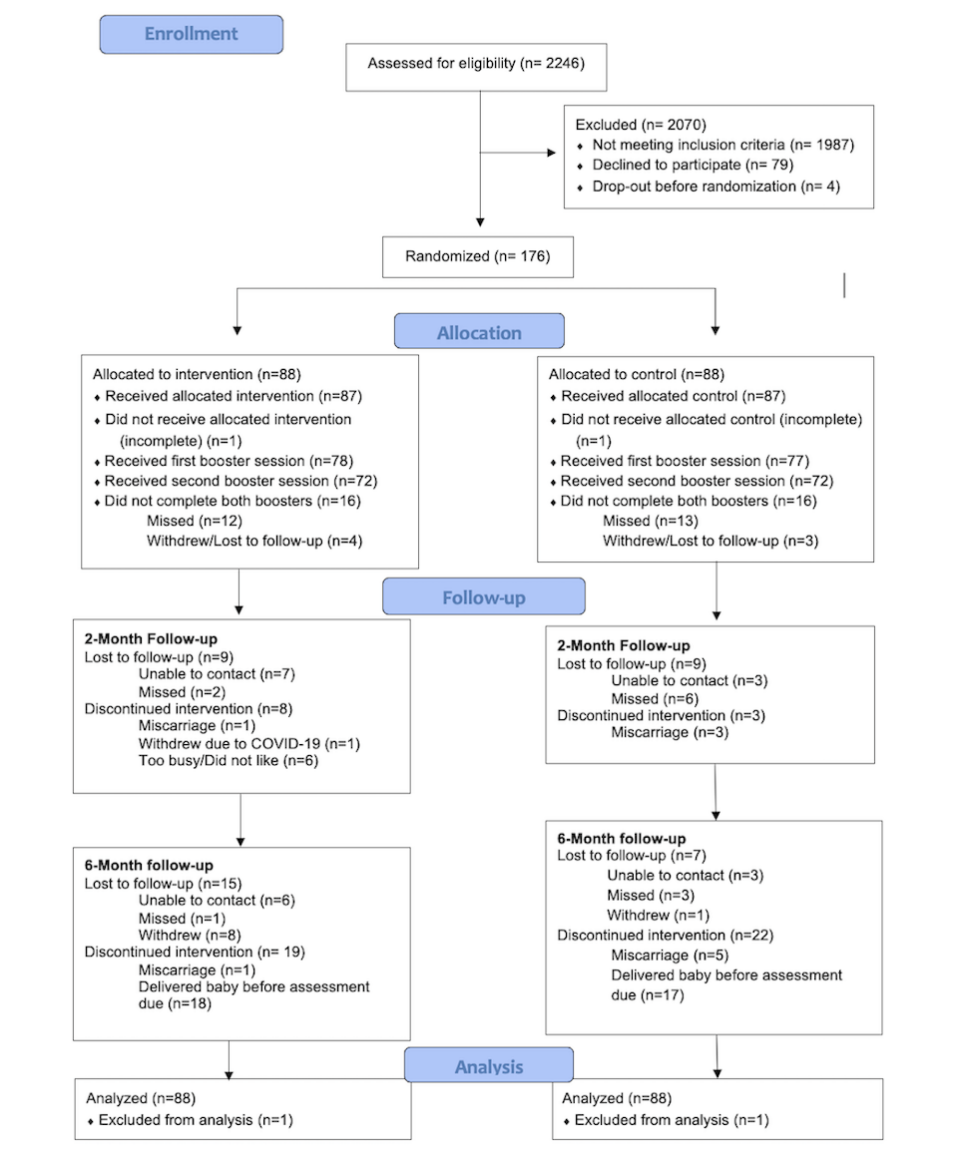
CONSORT (Consolidated Standards of Reporting Trial) flowchart.

A total of 8559 women were approached or contacted (through active and passive methods) during recruitment for the screening phase of the study (Figure 1, CONSORT [Consolidated Standards of Reporting Trials] flowchart). Of those approached, 6313 women (74%) declined the screener for reasons including being previously approached, too busy, not interested, or not eligible based on study criteria. Of the 2246 women assessed for eligibility, 2066 were excluded for reasons including not meeting study inclusion criteria, loss to follow-up, or withdrawing before enrollment. Overall, 180 women were enrolled for the randomized trial (8% of the women who were assessed for eligibility), 176 of them (97.7%) were randomized, and 174 completed both the baseline assessment and the intervention session.

Of the 88 women randomized to the intervention condition, 72 (82%) completed both booster sessions. Seventy women (80%) completed the 2-month follow-up assessment, and 47 women (53%) completed the 6-month follow-up assessment. Of the 88 women randomized to the control condition, 72 (82%) completed both booster sessions. Seventy-six women (86%) completed the 2-month follow-up assessment (antenatally), and 50 women (57%) completed the 6-month follow-up assessment (antenatally). Ten women (6%) withdrew from the study after enrollment (at varying time points), and 10 women (6%) were lost to follow-up and did not complete any follow-up assessments. A further 6 (3%) enrolled women became ineligible after study enrollment due to pregnancy loss.

We report participant characteristics in Table 1. In brief, our sample included a total of 176 pregnant cisgender women between the ages of 18-45, ≤21 weeks’ gestation (mean age 30.2, SD 5; mean gestational age 13.8 weeks, SD 4.45; 45/176, 25.6% Black; 17/176, 9.7% Latina; 41/176 23.3% single; 56/176, 31.8% receiving public assistance) reporting substance use and STI risk. There were significant differences in three of the characteristics between the control and intervention group, namely, “Family Income,” “Receive Public Assistance,” and “Current Marital Status”; see Table 1.

We found that our results did not differ when controlling for recruitment method and the method × arm × time interaction. We include in Table 1 the comparison of the three recruitment methods between the 2 groups (*P*=.99), which helps to explain why we did not include recruitment method as a covariate. We report that with remote and social media recruitment, as compared to in-person recruitment, we were more likely to recruit women reporting lower income (*P*=.02) and identifying as Latina (*P*=.04). With respect to predictors of loss to follow-up, participants who did not receive both boosters had 22.5 times higher odds of loss to follow-up compared to those who received both boosters, regardless of recruitment method (odds ratio 22.52, 95% CI 7.13-71.18; *P*<.001).

### Primary Outcomes: Efficacy of HCEM

#### Alcohol Use

**Figure 2:**
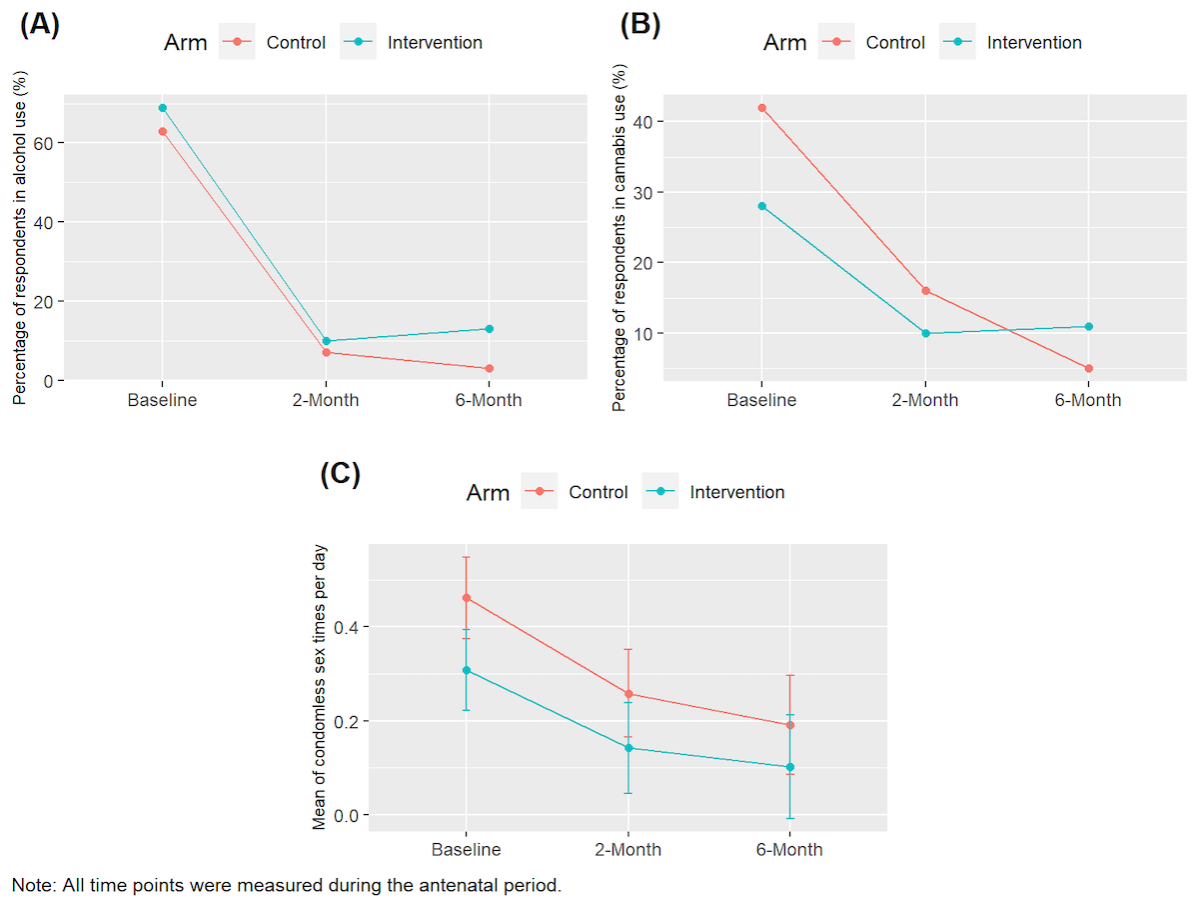
Panel plot showing all three outcomes together. All time points were measured during the antenatal period.

**Figure 3:**
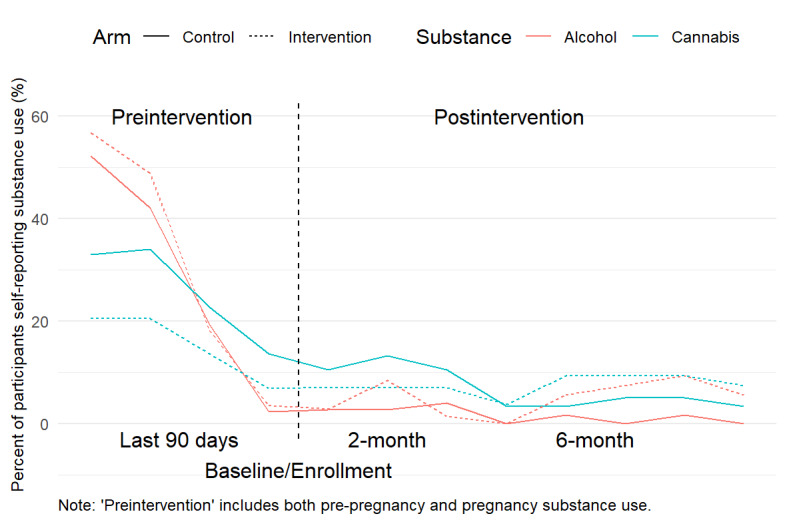
Substance use reductions before health check-up for expectant moms study enrollment.

Most of our sample reported low-frequency alcohol use. At baseline (which included both pre-pregnancy and pregnancy drinking), the average of standard drinks was 1.81 (SD 1.59; range 0.1-16), with a mode of 1 standard drink. Of all participants completing the baseline assessment, 65.9% (116/176) of participants reported at least one or more standard drinks of alcohol within the last 90 days (61/88, 69.3% HCEM vs 55/88, 62.5% control; *P*=.34; Figure 2A). Past month alcohol use at baseline was reported as 2.84% (5/176) overall (3.41% (3/88) HCEM vs 2.27% (2/88) control condition; *P*>.99), indicating a large reduction in use before enrolling in the study. At the 2-month follow-up, 8.2% (12/147) of participants (7/71, 9.9% HCEM vs 5/76, 6.6% control; *P*=.47) reported at least one drink, and 8% (9/113) reported at least one drink at the 6-month follow-up (7/54, 13%, HCEM vs 3.4%, 2/59 control; *P*=.08; Figure 3).

In the adjusted model, compared with baseline, the HCEM group had a 93% reduction at 2 months (adjusted odds ratio [AOR] 0.07, 95% CI 0.03-0.17; *P*<.001) and a 95% reduction at 6 months (AOR 0.05, 95% CI 0.02-0.12; *P*<.001). Similarly, the control group showed significant reductions: 94% at 2 months (AOR 0.06, 95% CI 0.02-0.18; *P*<.001) and 99% at 6 months (AOR 0.01, 95% CI 0-0.09; *P*<.001) (Table 2). However, there were no significant differences in the change pattern across the groups (overall time-by-group interaction, *P*=.16).

**Table 2:** Alcohol use regression results.

Parameters			AOR^a^ (95% CI)		*P* value
Age			0.98 (0.93-1.04)		.48
Marital (reference: legally married)					
	Living together but not married	0.51 (0.23-1.16)		.11	
	Single (never married or separated or divorced or widowed)	1.26 (0.63-2.49)		.51	
Arm (reference: control)					
	Baseline	1.29 (0.67-2.48)		.44	
	2-month follow-up	1.56 (0.49-4.98)		.45	
	6-month follow-up	4.93 (0.68-35.92)		.12	
Time (reference: baseline)					
	HCEM^b^: 2-month follow-up	0.07 (0.03-0.17)		<.001	
	HCEM: 6-month follow-up	0.05 (0.02-0.12)		<.001	
	Control: 2-month follow-up	0.06 (0.02-0.18)		<.001	
	Control: 6-month follow-up	0.01 (0-0.09)		<.001	
Arm * time					
	2-month follow-up	1.21 (0.32-4.55)		.78	
	6-month follow-up	3.82 (0.46-31.71)		.21	

^a^AOR: adjusted odds ratio.

^b^HCEM: health check-up for expectant moms.

#### Cannabis Use

Of all participants completing the baseline assessment (which included both pre-pregnancy and pregnancy), 35.2% (62/176) of participants reported cannabis use within the past 90 days (25/88, 28.4% HCEM condition vs 42% (37/88) control condition; *P*=.06; Figure 2B). Past month cannabis use at baseline reported as 10.2% (18/176) overall (6/88, 6.82% HCEM condition vs 12/88, 13.6% control condition; *P*=.14). At the 2-month follow-up assessment, 12.9% (19/147) reported cannabis use (7/71, 9.9% HCEM condition vs 12/76, 15.8% control condition; *P*=.28), and 8% (9/113) at the 6-month follow-up assessment (6/54, 11.1% HCEM condition vs 3/59, 5.1% control condition; *P*=.31; Figure 3).

Both the HCEM and control condition arms show significant reductions over time in cannabis use compared to baseline (Table 3). In the regression model adjusted for marital status, the HCEM group had a 71% reduction at 2 months (AOR 0.29, 95% CI 0.13-0.64; *P*=.002) and an 86% reduction at 6 months (AOR 0.14, 95% CI 0.06-0.37; *P*<.001). Similarly, the control group showed significant reductions: 71% at 2 months (AOR 0.29, 95% CI 0.14-0.61; *P*=.001) and 96% at 6 months (AOR 0.04, 95% CI 0.01-0.13; *P*<.001). However, the time-by-group interaction terms indicate no statistically significant differences in the reduction between the HCEM and control arms at either the 2-month (*P*=.98) or 6-month follow-up (*P*=.11). The *P* value for the overall time-by-arm interaction was estimated to be .06. Cannabis use showed a significantly decreasing trend with increasing age (*P*=.03). Participants who were legally married consumed significantly less cannabis compared to either the group living together or the group currently without a committed partner (both *P*s<.001) (Table 3).

**Table 3:** Cannabis use regression results.

Parameters			AOR^a^ (95% CI)		*P* value		
Age			0.94 (0.88-0.99)		.03		
Marital (reference: legally married)							
	Living together but not married	5.76 (2.27-14.67)		<.001			
	Single (never married or separated or divorced or widowed)	12.57 (5.51-28.68)		<.001			
Arm (reference: control)							
	Baseline	0.79 (0.37-1.69)		.55			
	2-month follow-up	0.78 (0.27-2.28)		.65			
	6-month follow-up	2.66 (0.64-11.13)		.18			
Time (reference: baseline)							
	HCEM^b^: 2-month follow-up	0.29 (0.13-0.64)		.002			
	HCEM: 6-month follow-up	0.14 (0.06-0.37)		<.001			
	Control: 2-month follow-up	0.29 (0.14-0.61)		.001			
	Control: 6-month follow-up	0.04 (0.01-0.13)		<.001			
Arm * time							
	2-month follow-up	0.99 (0.33-2.98)		.98			
	6-month follow-up	3.35 (0.76-14.74)		.11			

^a^AOR: adjusted odds ratio.

^b^HCEM: health check-up for expectant moms.

#### Comparison of Unprotected Sex Frequency Between Arms

At baseline, 99.4% (175/176) of participants reported engaging in condomless vaginal sex at compared with 87.8% (129/147) at 2-month follow-up assessment, and 84.1% (95/113) at 6-month follow-up assessment. Both groups demonstrated a decrease in the frequency over time (Figure 2C). In the adjusted model, this risky behavior was significantly lower in the intervention group both at the 2-month follow-up assessment (risk ratio [RR] 0.61, 95% CI: 0.46-0.82; *P*<.001), as well as the 6-month follow-up assessment (RR 0.58, 95% CI 0.38-0.88; *P*=.01) (Table 4). Compared with baseline, the HCEM group decreased by 50% at 2 months (RR 0.50, 95% CI 0.38-0.66; *P*<.001) and 64% at 6 months (RR 0.36, 95% CI 0.25-0.51; *P*<.001), while the control group decreased by 37% at 2 months (RR 0.63, 95% CI 0.50-0.80; *P*<.001) and 53% at 6 months (RR 0.47, 95% CI 0.34-0.66; *P*<.001). There were no statistically significant differences in the change pattern across the groups (overall time-by-group interaction, *P*=.47). As in the case of cannabis use, unprotected sex was least prevalent among the women who were legally married (Table 4).

**Table 4:** Unprotected sex frequency regression results.

Parameters		Rate ratios (95% CI)	*P* value
Age		1 (0.96-1.04)	.93
Marital (reference: legally married)			
	Living together but not married	1.84 (1.25-2.7)	.002
	Single (never married or separated or divorced or widowed)	1.78 (1.25-2.52)	.001
Arm (reference: control)			
	Baseline	0.77 (0.55-1.08)	.13
	2-month follow-up	0.61 (0.46-0.82)	<.001
	6-month follow-up	0.58 (0.38-0.88)	.01
Time (reference: baseline)			
	HCEM^a^: 2-month follow-up	0.5 (0.38-0.66)	<.001
	HCEM: 6-month follow-up	0.36 (0.25-0.51)	<.001
	Control: 2-month follow-up	0.63 (0.5-0.8)	<.001
	Control: 6-month follow-up	0.47 (0.34-0.66)	<.001
Arm * time			
	2-month follow-up	0.8 (0.55-1.14)	.22
	6-month follow-up	0.75 (0.46-1.22)	.24

^a^HCEM: health check-up for expectant moms.

## Discussion

The objective of this study was to test whether the web-based HCEM intervention reduced STI risk, alcohol, or drug use, more than an attention, time, and information-matched control condition in pregnant women seeking prenatal care. We screened a total of 2246 women, with 8% (180/2246) enrolled. Our completion rates were high, with 88% (155/176) of participants completing the first booster session and 82% (144/176) of participants completing the second booster session. We had a significant number of participants (63/176, 35%) miss their 6-month follow-up assessment largely due to giving birth before the assessment target date. Regarding the efficacy of the HCEM, there was a significant self-reported decrease in alcohol use among all participants in the month preceding study enrollment, which is consistent with earlier work that demonstrates spontaneous quitting and cessation rates. While we can infer from these reductions that the knowledge of their pregnancy status likely influenced self-change in their use, which is supported in previous work [71], our study did not formally assess when participants became aware of their pregnancy. Second, an intervention effect may have been impossible to observe given this substantial self-change. Third, the pandemic affected multiple aspects of our trial, including our study sample, which in turn may have impacted our outcomes [72]. Recent clinical trials report the impact of the SARS-CoV-2 pandemic on health outcomes. As a result, one clinical trial for chronic pain developed the PMC Coronavirus Pandemic Measure to help assess the moderation of treatment effects caused by the pandemic [73]. Fourth, a significant number of participants missed their 6-month assessment because they gave birth before the assessment target date, which resulted in only 65% of participants completing this follow-up assessment.

To our knowledge, the HCEM is the only theory-driven, technology-delivered brief intervention that simultaneously targets the co-occurring risks of alcohol or drug use and STIs during pregnancy. Furthermore, we include a control condition that was matched for time, attention, and information, and did not overlap with the actual HCEM. Given the broad continuum of cannabis use patterns from pre-pregnancy to postpartum, providing such an intervention during pregnancy - even to those reporting low to no current substance use - can have preventive effects that can extend throughout this period [74]. While not a main outcome, a particular strength of our study methodology was the inclusion of biological samples (hair or urine) for drug use. Unfortunately, we could not collect these samples from a significant proportion of our sample due to the pandemic restrictions (missing were 30% at baseline, 43% at 2-month follow-up, and 55% at 6-month follow-up), which affected our ability to include objective data for all participants. Additionally, our results mirror national reports of increases in prenatal cannabis use for young, unmarried pregnant women as compared to older, married pregnant women [75,76].

The limitations of our study include a relatively small sample size, which reduces the statistical power, a highly educated sample, a limited geographical sample in one midwestern state, and the inability to generalize our results to other pregnant individuals. Moreover, our sample reported very low frequency risk behaviors, especially alcohol use, during pregnancy. This may reflect our broad inclusion criteria for alcohol and drug use risk, and/or the nature of self-report in the disclosure of these behaviors during pregnancy. It is important to recognize that social desirability bias may have affected the outcomes, particularly during pregnancy when stigma and fears of disclosure can be significant [77,78]. Additionally, we did not formally assess the timepoint for participants’ knowledge of pregnancy and, therefore, cannot determine if their pregnancy status alone led to the observed reductions in the TLFB data. With respect to our self-reported outcome data, future trials could include an online version of the TLFB [79-81] as we recognize that interviews have been associated with underreporting of substance use [81]. Both conditions received informational brochures with facts about alcohol and drug use, STI risk, which may have impacted study outcomes. Furthermore, while we were able to conduct this clinical trial during the pandemic, it is important to acknowledge the possibility that the pandemic caused the study sample to be somewhat different from what it would have been otherwise, which might have impacted outcomes [72]. Additionally, while not a limitation of our study, we acknowledge that recreational cannabis use became legal in the state of Michigan during study recruitment and may have led to overall increases in use among our sample and the possibility of indirectly affecting the impact of the HCEM.

Prior studies have examined the potential role of psychosocial factors in either the continuation or cessation of alcohol and drug use when becoming pregnant. For example, one study of 1492 prenatal care patients from four urban clinics reported cessation rates of 87% for alcohol since learning of their pregnancy status, suggesting knowledge of fetal alcohol effects. The cessation rate for drug use was 55.6%. Predictors for both alcohol and drug use continuation included older age, current smoking, and lack of transportation. Pre-pregnancy alcohol use frequency, depression, and physical or sexual abuse were unique predictors of alcohol use continuation; race and ethnicity, and pre-pregnancy drug use frequency predicted continued drug use [82]. A study of the National Survey on Drug Use and Health data analyzed results from 1800 pregnant and 37,527 nonpregnant women and found that being White, unemployed, and possibly experiencing current psychopathology were more likely to report ongoing substance use during pregnancy [83]. Continued SBIs that integrate more than one focus (psychosocial concern or comorbidity) may better support continued substance use cessation during the pregnancy and postpartum period, particularly with high rates of relapse in the postpartum period, with one study citing 80% [84]. Extending prevention and support into the postpartum period is recommended for pregnant women with a recent history of drug use, as they are at greater risk for increased drug use in the initial months of postpartum [85]. Furthermore, the literature supports the superiority of targeting multiple health behaviors rather than just focusing on one behavior [86-88].

Technology-based interventions delivered during pregnancy are of critical importance for several reasons. First, we are experiencing an increase in maternity care deserts nationwide, as well as limited maternal health care clinicians and birthing facilities [42]. This complicates the picture for pregnant women needing specialized maternity care for substance use and/or mental health conditions [42]. Second, technology offers potential solutions for reducing fears of stigma and loss of confidentiality when disclosing sensitive information like substance use [43], intimate partner violence [89,90], and sexual risk behavior [39]. Related to these fears is the reality of legal repercussions for disclosing substance use during pregnancy [91,92]. This is especially a concern for low-income and minority women who are more likely to face legal consequences for disclosing substance use during pregnancy [42,93-95]. Third, web-based interventions circumvent transportation barriers, which is a problem when women either lack access to reliable transportation, and/or live hours away from maternal care facilities [42,96]. Finally, web-based interventions offer a cost-effective approach to reaching women, as they are accessible, self-directed programs that require little clinician time. Furthermore, the literature supports the effectiveness of web-based, or eHealth interventions in reducing substance use in pregnancy, and finds that it is a more equitable approach for addressing some of the barriers to care [43,97]. The reach and impact of web-based interventions are further magnified for pregnant women, as they have the potential to reduce harm to both mother and fetus, and can extend through the postpartum period.

Overall, our study examined the potential benefits of a web-based intervention seeking to reduce substance use and STI risk behaviors during pregnancy. While both conditions were associated with reductions in alcohol and drug use that, for the majority of participants, began in the month prior to study enrollment, an intervention effect may have been impossible to observe given substantial self-change. Additional factors include our small sample and the unique impacts of the SARS-CoV-2 pandemic on our study. Therefore, future studies should focus on women at higher risk of continued alcohol and/or cannabis use after knowing they are pregnant.

## Data Availability

Study participants were not asked and did not consent to have their data publicly available. Deidentified data reported in this manuscript are available by request to the senior author.

## Appendix

Multimedia Appendix 1 CONSORT-eHEALTH checklist (V 1.6.1).

## Abbreviations

ACOG: American College of Obstetricians
AOR: adjusted odds ratio
CDC: Centers for Disease Control and Prevention
CONSORT: Consolidated Standards of Reporting Trials
HCEM: health check-up for expectant moms
MI: motivational interviewing
RR: risk ratio
SBI: screening and brief intervention
STI: sexually transmitted infection
TLFB: timeline follow-back
