# Supplementary material for: A Web-Based Intervention for Reducing Sexually Transmitted Infections and Substance Use During Pregnancy: Randomized Controlled Trial
Source: J Med Internet Res. 2026 Jul 8;28:e95944. doi: 10.2196/95944 (PMC13392536; doi:10.2196/95944)
Supplement: Multimedia Appendix 1 [file jmir_v28i1e95944_app1.pdf]

CONSORT-EHEALTH (V 1.6.1) - Submission/Publication Form

The CONSORT-EHEALTH checklist is intended for authors of randomized trials evaluating web-based and Internet-based applications/interventions, including mobile interventions, electronic games (incl multiplayer games), social media, certain telehealth applications, and other interactive and/or networked electronic applications. Some of the items (e.g. all subitems under item 5 - description of the intervention) may also be applicable for other study designs.

The goal of the CONSORT EHEALTH checklist and guideline is to be
a) a guide for reporting for authors of RCTs,
b) to form a basis for appraisal of an ehealth trial (in terms of validity)

CONSORT-EHEALTH items/subitems are MANDATORY reporting items for studies published in the Journal of Medical Internet Research and other journals / scientific societies endorsing the checklist.

Items numbered 1., 2., 3., 4a., 4b etc are original CONSORT or CONSORT-NPT (non-pharmacologic treatment) items.
Items with Roman numerals (i., ii, iii, iv etc.) are CONSORT-EHEALTH extensions/clarifications.

As the CONSORT-EHEALTH checklist is still considered in a formative stage, we would ask that you also RATE ON A SCALE OF 1-5 how important/useful you feel each item is FOR THE PURPOSE OF THE CHECKLIST and reporting guideline (optional).

Mandatory reporting items are marked with a red *.
In the textboxes, either copy & paste the relevant sections from your manuscript into this form - please include any quotes from your manuscript in QUOTATION MARKS,
or answer directly by providing additional information not in the manuscript, or elaborating on why the item was not relevant for this study.

YOUR ANSWERS WILL BE PUBLISHED AS A SUPPLEMENTARY FILE TO YOUR PUBLICATION IN JMIR AND ARE CONSIDERED PART OF YOUR PUBLICATION (IF ACCEPTED).
Please fill in these questions diligently. Information will not be copyedited, so please use proper spelling and grammar, use correct capitalization, and avoid abbreviations.

DO NOT FORGET TO SAVE AS PDF _AND_ CLICK THE SUBMIT BUTTON SO YOUR ANSWERS ARE IN OUR DATABASE !!!

Citation Suggestion (if you append the pdf as Appendix we suggest to cite this paper in the caption):
Eysenbach G, CONSORT-EHEALTH Group

CONSORT-EHEALTH: Improving and Standardizing Evaluation Reports of Web-based and Mobile Health Interventions
J Med Internet Res 2011;13(4):e126
URL: http://www.jmir.org/2011/4/e126/
doi: 10.2196/jmir.1923
PMID: 22209829

kristina.countryman@gmail.com Switch account

Draft saved

Not shared

* Indicates required question

Your name *

First Last

Golfo Tzilos Wernette

Primary Affiliation (short), City, Country *

University of Toronto, Toronto, Canada

University of Michigan, Ann Arbor, MI, USA

Your e-mail address *

abc@gmail.com

gtzilos@med.umich.edu

Title of your manuscript *

Provide the (draft) title of your manuscript.

A Web-Based Intervention for Reducing Sexually Transmitted Infections and Substance Use During Pregnancy: A Randomized Controlled Trial

Name of your App/Software/Intervention *

If there is a short and a long/alternate name, write the short name first and add the long name in brackets.

HCEM (Health Check-up for Expectant Moms)

Evaluated Version (if any)

e.g. "V1", "Release 2017-03-01", "Version 2.0.27913"

Your answer

Language(s) *

What language is the intervention/app in? If multiple languages are available, separate by comma (e.g. "English, French")

English

URL of your Intervention Website or App

e.g. a direct link to the mobile app on app in appstore (itunes, Google Play), or URL of the website. If the intervention is a DVD or hardware, you can also link to an Amazon page.

Your answer

URL of an image/screenshot (optional)

Your answer

Accessibility *

Can an enduser access the intervention presently?

- ◯ access is free and open
- ◯ access only for special usergroups, not open
- ◯ access is open to everyone, but requires payment/subscription/in-app purchases
- ◉ app/intervention no longer accessible
- ◯ Other:

Primary Medical Indication/Disease/Condition *

e.g. "Stress", "Diabetes", or define the target group in brackets after the condition, e.g. "Autism (Parents of children with)", "Alzheimers (Informal Caregivers of)"

"Substance Use" "Pregnancy" "Sexually Transm

Primary Outcomes measured in trial *

comma-separated list of primary outcomes reported in the trial

Reductions in alcohol/drug use; Reductions in

Secondary/other outcomes

Are there any other outcomes the intervention is expected to affect?

Your answer

Recommended "Dose" *

What do the instructions for users say on how often the app should be used?

- ◯ Approximately Daily
- ◯ Approximately Weekly
- ◯ Approximately Monthly
- ◯ Approximately Yearly
- ◯ "as needed"
- ◉ Other: One initial 40-minute session/dose with two shorter (10-15 min.) boos

Approx. Percentage of Users (starters) still using the app as recommended after 3 months *

- ( ) unknown / not evaluated
- ( ) 0-10%
- ( ) 11-20%
- ( ) 21-30%
- ( ) 31-40%
- ( ) 41-50%
- ( ) 51-60%
- ( ) 61-70%
- ( ) 71%-80%
- ( ) 81-90%
- ( ) 91-100%
- (•) Other: Not applicable to current study

Overall, was the app/intervention effective? *

- ( ) yes: all primary outcomes were significantly better in intervention group vs control
- ( ) partly: SOME primary outcomes were significantly better in intervention group vs control
- ( ) no statistically significant difference between control and intervention
- ( ) potentially harmful: control was significantly better than intervention in one or more outcomes
- ( ) inconclusive: more research is needed
- ( ) Other:

Article Preparation Status/Stage *

At which stage in your article preparation are you currently (at the time you fill in this form)

- ○ not submitted yet - in early draft status
- ◉ not submitted yet - in late draft status, just before submission
- ○ submitted to a journal but not reviewed yet
- ○ submitted to a journal and after receiving initial reviewer comments
- ○ submitted to a journal and accepted, but not published yet
- ○ published
- ○ Other:

Journal *

If you already know where you will submit this paper (or if it is already submitted), please provide the journal name (if it is not JMIR, provide the journal name under "other")

- ○ not submitted yet / unclear where I will submit this
- ◉ Journal of Medical Internet Research (JMIR)
- ○ JMIR mHealth and UHealth
- ○ JMIR Serious Games
- ○ JMIR Mental Health
- ○ JMIR Public Health
- ○ JMIR Formative Research
- ○ Other JMIR sister journal
- ○ Other:

Is this a full powered effectiveness trial or a pilot/feasibility trial? *

- ○ Pilot/feasibility
- ◉ Fully powered

Manuscript tracking number *

If this is a JMIR submission, please provide the manuscript tracking number under "other" (The ms tracking number can be found in the submission acknowledgement email, or when you login as author in JMIR. If the paper is already published in JMIR, then the ms tracking number is the four-digit number at the end of the DOI, to be found at the bottom of each published article in JMIR)

- ◉ no ms number (yet) / not (yet) submitted to / published in JMIR
- ○ Other:

TITLE AND ABSTRACT

1a) TITLE: Identification as a randomized trial in the title

1a) Does your paper address CONSORT item 1a? *

I.e does the title contain the phrase "Randomized Controlled Trial"? (if not, explain the reason under "other")

- ◉ yes
- ○ Other:

1a-i) Identify the mode of delivery in the title

Identify the mode of delivery. Preferably use “web-based” and/or “mobile” and/or “electronic game” in the title. Avoid ambiguous terms like “online”, “virtual”, “interactive”. Use “Internet-based” only if Intervention includes non-web-based Internet components (e.g. email), use “computer-based” or “electronic” only if offline products are used. Use “virtual” only in the context of “virtual reality” (3-D worlds). Use “online” only in the context of “online support groups”. Complement or substitute product names with broader terms for the class of products (such as “mobile” or “smart phone” instead of “iphone”), especially if the application runs on different platforms.

	1	2	3	4	5	
subitem not at all important	○	○	○	○	◉	essential

Clear selection

Does your paper address subitem 1a-i? *

Copy and paste relevant sections from manuscript title (include quotes in quotation marks "like this" to indicate direct quotes from your manuscript), or elaborate on this item by providing additional information not in the ms, or briefly explain why the item is not applicable/relevant for your study

"A Web-Based Intervention for Reducing Sexually Transmitted Infections and Substance Use During Pregnancy: A Randomized Controlled Trial"

1a-ii) Non-web-based components or important co-interventions in title

Mention non-web-based components or important co-interventions in title, if any (e.g., “with telephone support”).

	1	2	3	4	5	
subitem not at all important	○	○	○	○	○	essential

Does your paper address subitem 1a-ii?

Copy and paste relevant sections from manuscript title (include quotes in quotation marks "like this" to indicate direct quotes from your manuscript), or elaborate on this item by providing additional information not in the ms, or briefly explain why the item is not applicable/relevant for your study

Your answer

1a-iii) Primary condition or target group in the title

Mention primary condition or target group in the title, if any (e.g., “for children with Type I Diabetes”) Example: A Web-based and Mobile Intervention with Telephone Support for Children with Type I Diabetes: Randomized Controlled Trial

	1	2	3	4	5	
subitem not at all important	○	○	○	○	◉	essential

Clear selection

Does your paper address subitem 1a-iii? *

Copy and paste relevant sections from manuscript title (include quotes in quotation marks "like this" to indicate direct quotes from your manuscript), or elaborate on this item by providing additional information not in the ms, or briefly explain why the item is not applicable/relevant for your study

"A Web-Based Intervention for Reducing Sexually Transmitted Infections and Substance Use During Pregnancy: A Randomized Controlled Trial"

1b) ABSTRACT: Structured summary of trial design, methods, results, and conclusions

NPT extension: Description of experimental treatment, comparator, care providers, centers, and blinding status.

1b-i) Key features/functionalities/components of the intervention and comparator in the METHODS section of the ABSTRACT

Mention key features/functionalities/components of the intervention and comparator in the abstract. If possible, also mention theories and principles used for designing the site. Keep in mind the needs of systematic reviewers and indexers by including important synonyms. (Note: Only report in the abstract what the main paper is reporting. If this information is missing from the main body of text, consider adding it)

	1	2	3	4	5	
subitem not at all important	○	○	○	○	○	essential

Does your paper address subitem 1b-i? *

Copy and paste relevant sections from the manuscript abstract (include quotes in quotation marks "like this" to indicate direct quotes from your manuscript), or elaborate on this item by providing additional information not in the ms, or briefly explain why the item is not applicable/relevant for your study

"We recruited a sample of 176 pregnant women (all were at risk for alcohol/drug use or STIs during pregnancy) from clinics and using social media campaigns (Facebook® and Instagram®) in the state of Michigan and randomized them to the Motivational Interviewing (MI)-consistent HCEM intervention or to an attention, time, and information-matched control condition delivered using the same technology platform. We followed these women at 2- and 6- months after the initial intervention visit. Primary outcomes included self-report assessment of alcohol, drug/cannabis use, and unprotected sexual occasions during pregnancy."

1b-ii) Level of human involvement in the METHODS section of the ABSTRACT

Clarify the level of human involvement in the abstract, e.g., use phrases like “fully automated” vs. “therapist/nurse/care provider/physician-assisted” (mention number and expertise of providers involved, if any). (Note: Only report in the abstract what the main paper is reporting. If this information is missing from the main body of text, consider adding it)

	1	2	3	4	5	
subitem not at all important	○	○	○	○	○	essential

Does your paper address subitem 1b-ii?

Copy and paste relevant sections from the manuscript abstract (include quotes in quotation marks "like this" to indicate direct quotes from your manuscript), or elaborate on this item by providing additional information not in the ms, or briefly explain why the item is not applicable/relevant for your study

"Objective: We tested whether an innovative, theory-driven, technology-delivered and fully automated brief intervention (single session plus two booster sessions), the Health Check-up for Expectant Moms (HCEM), reduced condomless sex/STI risk, alcohol, or drug use, compared to a control condition in pregnant women seeking prenatal care."

1b-iii) Open vs. closed, web-based (self-assessment) vs. face-to-face assessments in the METHODS section of the ABSTRACT

Mention how participants were recruited (online vs. offline), e.g., from an open access website or from a clinic or a closed online user group (closed usergroup trial), and clarify if this was a purely web-based trial, or there were face-to-face components (as part of the intervention or for assessment). Clearly say if outcomes were self-assessed through questionnaires (as common in web-based trials). Note: In traditional offline trials, an open trial (open-label trial) is a type of clinical trial in which both the researchers and participants know which treatment is being administered. To avoid confusion, use “blinded” or “unblinded” to indicated the level of blinding instead of “open”, as “open” in web-based trials usually refers to “open access” (i.e. participants can self-enrol). (Note: Only report in the abstract what the main paper is reporting. If this information is missing from the main body of text, consider adding it)

	1	2	3	4	5	
subitem not at all important	○	○	○	○	○	essential

Does your paper address subitem 1b-iii?

Copy and paste relevant sections from the manuscript abstract (include quotes in quotation marks "like this" to indicate direct quotes from your manuscript), or elaborate on this item by providing additional information not in the ms, or briefly explain why the item is not applicable/relevant for your study

"We recruited a sample of 176 pregnant women (all were at risk for alcohol/drug use or STIs during pregnancy) from clinics and using social media campaigns (Facebook® and Instagram®) in the state of Michigan and randomized them to the Motivational Interviewing (MI)-consistent HCEM intervention or to an attention, time, and information-matched control condition delivered using the same technology platform. We followed these women at 2- and 6- months after the initial intervention visit. Primary outcomes included self-report assessment of alcohol, drug/cannabis use, and unprotected sexual occasions during pregnancy."

1b-iv) RESULTS section in abstract must contain use data

Report number of participants enrolled/assessed in each group, the use/uptake of the intervention (e.g., attrition/adherence metrics, use over time, number of logins etc.), in addition to primary/secondary outcomes. (Note: Only report in the abstract what the main paper is reporting. If this information is missing from the main body of text, consider adding it)

	1	2	3	4	5	
subitem not at all important	○	○	○	○	○	essential

Does your paper address subitem 1b-iv?

Copy and paste relevant sections from the manuscript abstract (include quotes in quotation marks "like this" to indicate direct quotes from your manuscript), or elaborate on this item by providing additional information not in the ms, or briefly explain why the item is not applicable/relevant for your study

"Results: A total of 88 women were randomized to the intervention, and 88 to the control condition. Cannabis use was the most prevalent substance reported during pregnancy; a total of 35.2% reported recent use (within the last ninety days) at baseline."

1b-v) CONCLUSIONS/DISCUSSION in abstract for negative trials

Conclusions/Discussions in abstract for negative trials: Discuss the primary outcome - if the trial is negative (primary outcome not changed), and the intervention was not used, discuss whether negative results are attributable to lack of uptake and discuss reasons. (Note: Only report in the abstract what the main paper is reporting. If this information is missing from the main body of text, consider adding it)

	1	2	3	4	5	
subitem not at all important	○	○	○	○	○	essential

Does your paper address subitem 1b-v?

Copy and paste relevant sections from the manuscript abstract (include quotes in quotation marks "like this" to indicate direct quotes from your manuscript), or elaborate on this item by providing additional information not in the ms, or briefly explain why the item is not applicable/relevant for your study

"Conclusion: There are many potential benefits of a technology-delivered approach to support the behavioral health of pregnant women in a private and convenient way. Our sample was largely low-risk and as such, an intervention effect may have been impossible to observe given substantial self-change. Future trials are needed to examine efficacy in other samples of pregnant women with higher risk of current substance use."

INTRODUCTION

2a) In INTRODUCTION: Scientific background and explanation of rationale

2a-i) Problem and the type of system/solution

Describe the problem and the type of system/solution that is object of the study: intended as stand-alone intervention vs. incorporated in broader health care program? Intended for a particular patient population? Goals of the intervention, e.g., being more cost-effective to other interventions, replace or complement other solutions? (Note: Details about the intervention are provided in “Methods” under 5)

	1	2	3	4	5	
subitem not at all important	○	○	○	○	○	essential

Does your paper address subitem 2a-i? *

Copy and paste relevant sections from the manuscript (include quotes in quotation marks "like this" to indicate direct quotes from your manuscript), or elaborate on this item by providing additional information not in the ms, or briefly explain why the item is not applicable/relevant for your study

"Substance use during pregnancy is associated with several negative consequences for both the pregnant woman and the developing fetus, including increasing the incidence of STIs, postpartum depression (PPD), significant fetal and neonatal complications. Among pregnant women who use substances, the co-use of other substances among pregnant women is common; 38% of pregnant women who report current alcohol use also report use of one or more other substances (mostly tobacco and cannabis).7 Cannabis is the most frequently used drug during pregnancy, and several recent meta-analyses show that newborns with exposure to cannabis in utero were more likely to be born pre-term, have a low birth weight, and require neonatal intensive care compared to newborns without exposure. Alcohol use during pregnancy can lead to life-long adverse effects for the infant, including Fetal Alcohol Spectrum Disorders (FASD). There has been a dramatic five-fold national increase in opioid use during pregnancy, leading to a surge in the incidence of neonatal abstinence syndrome (NAS), a postnatal drug withdrawal syndrome that includes central nervous system irritability.

STIs remain at record highs in the United States, and STI risk continues to be a critical and costly health problem for women, especially for pregnant women who can transmit infections to their babies. STIs can negatively impact both the woman and baby and can result in premature birth, low birthweight, and fetal death. A pregnant woman is at risk of transmitting certain STIs (like HIV) to her infant during pregnancy, delivery, or breastfeeding, especially if untreated. Importantly, substance use and risky sexual behavior, together, contribute significantly to the acquisition of STIs, and this is especially concerning in vulnerable populations including during pregnancy."

AND

"Because the majority (97%) of pregnant women receive at least some prenatal care, the Centers for Disease Control and Prevention (CDC) and the American College of Obstetricians (ACOG) have identified the perinatal period as an urgent time for STI screening, prevention and subsequent behavior change, including substance use. Due to increased use nationwide, the ACOG recently issued a strong recommendation specifically for universal cannabis use screening during pregnancy through self-report rather than drug testing to prevent racial disparities (as significant racial disparities in drug testing are well documented). Further, postpartum women are vulnerable to increased risk of STIs associated with high-risk sexual behaviors, including inconsistent condom use, alcohol use (with national data suggesting prevalence rates as high as 30-49%), and drug use (with prevalence rates in the range of 4-9%). To optimize postpartum care, ACOG has recommended proactively addressing these risky health behaviors during pregnancy, given that as many as 40% of women in the U.S. do not receive postpartum care. Intervening on health concerns during a natural life event, such as pregnancy, has been shown to be an ideal time to introduce behavior change, as women may be more motivated to adopt healthier behaviors for the well-being of the child.

To this end, technology-delivered screening and brief intervention (SBI) is a confidential, self-directed, low-cost intervention that has several notable strengths for working with women in the perinatal period. The use of computer-assisted SBI for the disclosure of STI/HIV risk has been empirically supported in terms of encouraging the disclosure of sexual risk-taking and additional STI testing. This is of particular importance because web-based interventions have the potential to overcome known barriers to seeking care among pregnant women such as fear of disclosure and stigma. Furthermore, a recent review [illegible]

suggests that the use of electronic screening, brief intervention, or referral to treatment (e-SBIRT) for substance use may be an effective and more equitable approach for overcoming traditional barriers to care that prevent women with these risk factors from receiving treatment during pregnancy.
The Health Check-Up for Expectant Moms (HCEM) is an innovative, theory-driven, technology-delivered brief intervention –– a single-session plus two booster sessions – designed to reduce alcohol/drug use and risk for STIs during pregnancy. In the HCEM pilot RCT, we found very high ratings of acceptability (between 6.3 and 6.8 on a 1-7 scale) in our sample of 50 pregnant women, and noted that the HCEM was associated with significantly larger reduction (54%) in any cannabis or alcohol use during pregnancy compared with participants in the control group (16%; P=.015). To our knowledge, apart from our pilot work, no other studies have assessed web-based STI prevention interventions exclusively in non-treatment seeking pregnant women at risk for substance use. Building upon this pilot work, we report the results of our randomized controlled trial testing whether the HCEM reduced condomless sex/STI risk, alcohol/drug use, more than a control condition in a larger, more diverse sample of pregnant women seeking prenatal care."

2a-ii) Scientific background, rationale: What is known about the (type of) system

Scientific background, rationale: What is known about the (type of) system that is the object of the study (be sure to discuss the use of similar systems for other conditions/diagnoses, if appropiate), motivation for the study, i.e. what are the reasons for and what is the context for this specific study, from which stakeholder viewpoint is the study performed, potential impact of findings [2]. Briefly justify the choice of the comparator.

	1	2	3	4	5	
subitem not at all important	○	○	○	○	○	essential

Does your paper address subitem 2a-ii? *

Copy and paste relevant sections from the manuscript (include quotes in quotation marks "like this" to indicate direct quotes from your manuscript), or elaborate on this item by providing additional information not in the ms, or briefly explain why the item is not applicable/relevant for your study

"To this end, technology-delivered screening and brief intervention (SBI) is a confidential, self-directed, low-cost intervention that has several notable strengths for working with women in the perinatal period. The use of computer-assisted SBI for the disclosure of STI/HIV risk has been empirically supported in terms of encouraging the disclosure of sexual risk-taking and additional STI testing.This is of particular importance because web-based interventions have the potential to overcome known barriers to seeking care among pregnant women such as fear of disclosure and stigma. Furthermore, a recent review suggests that the use of electronic screening, brief intervention, or referral to treatment (e-SBIRT) for substance use may be an effective and more equitable approach for overcoming traditional barriers to care that prevent women with these risk factors from receiving treatment during pregnancy.
The Health Check-Up for Expectant Moms (HCEM) is an innovative, theory-driven, technology-delivered brief intervention –– a single-session plus two booster sessions – designed to reduce alcohol/drug use and risk for STIs during pregnancy. In the HCEM pilot RCT, we found very high ratings of acceptability (between 6.3 and 6.8 on a 1-7 scale) in our sample of 50 pregnant women, and noted that the HCEM was associated with significantly larger reduction (54%) in any cannabis or alcohol use during pregnancy compared with participants in the control group (16%; P=.015). To our knowledge, apart from our pilot work, no other studies have assessed web-based STI prevention interventions exclusively in non-treatment seeking pregnant women at risk for substance use. Building upon this pilot work, we report the results of our randomized controlled trial testing whether the HCEM reduced condomless sex/STI risk, alcohol/drug use, more than a control condition in a larger, more diverse sample of pregnant women seeking prenatal care."

2b) In INTRODUCTION: Specific objectives or hypotheses

Does your paper address CONSORT subitem 2b? *

Copy and paste relevant sections from the manuscript (include quotes in quotation marks "like this" to indicate direct quotes from your manuscript), or elaborate on this item by providing additional information not in the ms, or briefly explain why the item is not applicable/relevant for your study

"Building upon this pilot work, we report the results of our randomized controlled trial testing whether the HCEM reduced condomless sex/STI risk, alcohol/drug use, more than a control condition in a larger, more diverse sample of pregnant women seeking prenatal care."

METHODS

3a) Description of trial design (such as parallel, factorial) including allocation ratio

Does your paper address CONSORT subitem 3a? *

Copy and paste relevant sections from the manuscript (include quotes in quotation marks "like this" to indicate direct quotes from your manuscript), or elaborate on this item by providing additional information not in the ms, or briefly explain why the item is not applicable/relevant for your study

"Using simple randomization, we computer randomized 176 pregnant women who met study inclusion criteria into the HCEM intervention (n = 88) or an attention, time, and information-matched control (n=88) condition."

3b) Important changes to methods after trial commencement (such as eligibility criteria), with reasons

Does your paper address CONSORT subitem 3b? *

Copy and paste relevant sections from the manuscript (include quotes in quotation marks "like this" to indicate direct quotes from your manuscript), or elaborate on this item by providing additional information not in the ms, or briefly explain why the item is not applicable/relevant for your study

"This was a change to our original (pre-COVID-19) inclusion criteria, where STI risk behavior was measured solely by self-reported recent (within the last 30 days) condomless vaginal sex (substance use risk criteria was the same). Due to participant feedback and the overall low rate of STI risk behavior observed in the data up to that point, we reviewed empirically supported methodology and aligned with the CDC recommendations to increase the threshold for the STI risk behavior eligibility."

3b-i) Bug fixes, Downtimes, Content Changes

Bug fixes, Downtimes, Content Changes: ehealth systems are often dynamic systems. A description of changes to methods therefore also includes important changes made on the intervention or comparator during the trial (e.g., major bug fixes or changes in the functionality or content) (5-iii) and other “unexpected events” that may have influenced study design such as staff changes, system failures/downtimes, etc. [2].

	1	2	3	4	5	
subitem not at all important	○	○	○	○	○	essential

Does your paper address subitem 3b-i?

Copy and paste relevant sections from the manuscript (include quotes in quotation marks "like this" to indicate direct quotes from your manuscript), or elaborate on this item by providing additional information not in the ms, or briefly explain why the item is not applicable/relevant for your study

"Initial participant recruitment took place in-person at family medicine and obstetrician/gynecology clinics between April 2019 and March 2020 (n = 81). Due to the COVID-19 pandemic, new recruitment stopped until August 2020, to allow time to make the necessary protocol and software changes for complete remote delivery (see previous paper for more detail)."

4a) Eligibility criteria for participants

Does your paper address CONSORT subitem 4a? *

Copy and paste relevant sections from the manuscript (include quotes in quotation marks "like this" to indicate direct quotes from your manuscript), or elaborate on this item by providing additional information not in the ms, or briefly explain why the item is not applicable/relevant for your study

"Specifically, inclusion criteria included a combination of questions assessing risk for substance use and health behaviors placing women at risk for STIs. Risk of substance use during pregnancy was determined using a combination of 3 substance use screeners validated for use during pregnancy including, the Substance Use Profile-Pregnancy (SURP-P) (3 items; one or more affirmative responses indicate a positive screen in low-risk populations), National Institute on Drug Abuse (NIDA) Quick Screen V.1/Modified-ASSIST V.2 (2 items; one or more affirmative responses indicates risk), and the Tolerance, Annoyed, Cut down, Eye opener (T-ACE) (4-items; T is 2 points and A,C, E are 1 point each. 2 points or more considered positive screen). To meet the substance use risk criteria for HCEM, women had to screen positive on at least one of these validated screeners above. Eligibility for STI risk behavior included recent (within the last 30 days) condomless vaginal sex, and at least one of the following: 1) multiple sexual partners within the last 6 months; 2) a new sexual partner since becoming pregnant; or 3) uncertainty of current partner's monogamy. This was a change to our original (pre-COVID-19) inclusion criteria, where STI risk behavior was measured solely by self-reported recent (within the last 30 days) condomless vaginal sex (substance use risk criteria was the same). Due to participant feedback and the overall low rate of STI risk behavior observed in the data up to that point, we reviewed empirically supported methodology and aligned with the CDC recommendations to increase the threshold for the STI risk behavior eligibility."

4a-i) Computer / Internet literacy

Computer / Internet literacy is often an implicit "de facto" eligibility criterion - this should be explicitly clarified.

	1	2	3	4	5	
subitem not at all important	○	○	○	○	○	essential

Does your paper address subitem 4a-i?

Copy and paste relevant sections from the manuscript (include quotes in quotation marks "like this" to indicate direct quotes from your manuscript), or elaborate on this item by providing additional information not in the ms, or briefly explain why the item is not applicable/relevant for your study

"Because participation in this study required a smart phone or computer/device with internet access, computer and internet literacy was a “de facto” eligibility criteria."

4a-ii) Open vs. closed, web-based vs. face-to-face assessments:

Open vs. closed, web-based vs. face-to-face assessments: Mention how participants were recruited (online vs. offline), e.g., from an open access website or from a clinic, and clarify if this was a purely web-based trial, or there were face-to-face components (as part of the intervention or for assessment), i.e., to what degree got the study team to know the participant. In online-only trials, clarify if participants were quasi-anonymous and whether having multiple identities was possible or whether technical or logistical measures (e.g., cookies, email confirmation, phone calls) were used to detect/prevent these.

	1	2	3	4	5	
subitem not at all important	○	○	○	○	○	essential

Does your paper address subitem 4a-ii? *

Copy and paste relevant sections from the manuscript (include quotes in quotation marks "like this" to indicate direct quotes from your manuscript), or elaborate on this item by providing additional information not in the ms, or briefly explain why the item is not applicable/relevant for your study

"Participants completed the assessment and intervention or control sessions in the clinic on the study iPad (pre-COVID) or on a personal smartphone or computer/device through a web link to the study website (post-COVID). Booster sessions were emailed to participants to be completed independently using a web link to the study website both pre- and post-COVID-19. Reminders by phone/text/email to complete booster sessions were sent as needed until the 2-month follow-up visit, after which booster sessions were no longer available to complete. Whether in-person or remote, a research study team member was available (during regular business hours) to help participants troubleshoot any technical problems. All participant contact (call/text/email) was conducted using university-secured and encrypted websites to ensure participant confidentiality and safety."

4a-iii) Information giving during recruitment

Information given during recruitment. Specify how participants were briefed for recruitment and in the informed consent procedures (e.g., publish the informed consent documentation as appendix, see also item X26), as this information may have an effect on user self-selection, user expectation and may also bias results.

	1	2	3	4	5	
subitem not at all important	○	○	○	○	○	essential

Does your paper address subitem 4a-iii?

Copy and paste relevant sections from the manuscript (include quotes in quotation marks "like this" to indicate direct quotes from your manuscript), or elaborate on this item by providing additional information not in the ms, or briefly explain why the item is not applicable/relevant for your study

"Study details at recruitment were limited; the study was simply referred to as a 'healthy pregnancy' study, with full details reviewed after a participant was deemed eligible."

4b) Settings and locations where the data were collected

Does your paper address CONSORT subitem 4b? *

Copy and paste relevant sections from the manuscript (include quotes in quotation marks "like this" to indicate direct quotes from your manuscript), or elaborate on this item by providing additional information not in the ms, or briefly explain why the item is not applicable/relevant for your study

"Initial participant recruitment took place in-person at family medicine and obstetrician/gynecology clinics between April 2019 and March 2020 (n = 81). Due to the COVID-19 pandemic, new recruitment stopped until August 2020, to allow time to make the necessary protocol and software changes for complete remote delivery (see previous paper for more detail). Remote recruitment began in August 2020 and ended in September 2023 (n = 95). Remote recruitment included calling/texting/emailing existing clinic patients and social media advertising (Facebook® and Instagram®) throughout the state of Michigan."

AND

"Participants completed the assessment and intervention or control sessions in the clinic on the study iPad (pre-COVID) or on a personal smartphone or computer/device through a web link to the study website (post-COVID). Booster sessions were emailed to participants to be completed independently using a web link to the study website both pre- and post-COVID-19."

4b-i) Report if outcomes were (self-)assessed through online questionnaires

Clearly report if outcomes were (self-)assessed through online questionnaires (as common in web-based trials) or otherwise.

	1	2	3	4	5	
subitem not at all important	○	○	○	○	○	essential

Does your paper address subitem 4b-i? *

Copy and paste relevant sections from the manuscript (include quotes in quotation marks "like this" to indicate direct quotes from your manuscript), or elaborate on this item by providing additional information not in the ms, or briefly explain why the item is not applicable/relevant for your study

"Participants completed an approximately 25-minute assessment session before starting the intervention or control session. The assessment battery was designed to minimize assessment with the time, attention, and information-matched control group to account for concerns regarding the motivational properties of assessment. All of the self-report measures have good psychometric properties:...."

4b-ii) Report how institutional affiliations are displayed

Report how institutional affiliations are displayed to potential participants [on ehealth media], as affiliations with prestigious hospitals or universities may affect volunteer rates, use, and reactions with regards to an intervention.(Not a required item – describe only if this may bias results)

	1	2	3	4	5	
subitem not at all important	○	○	○	○	○	essential

Does your paper address subitem 4b-ii?

Copy and paste relevant sections from the manuscript (include quotes in quotation marks "like this" to indicate direct quotes from your manuscript), or elaborate on this item by providing additional information not in the ms, or briefly explain why the item is not applicable/relevant for your study

Your answer

5) The interventions for each group with sufficient details to allow replication, including how and when they were actually administered

5-i) Mention names, credential, affiliations of the developers, sponsors, and owners

Mention names, credential, affiliations of the developers, sponsors, and owners [6] (if authors/evaluators are owners or developer of the software, this needs to be declared in a “Conflict of interest” section or mentioned elsewhere in the manuscript).

	1	2	3	4	5	
subitem not at all important	○	○	○	○	○	essential

Does your paper address subitem 5-i?

Copy and paste relevant sections from the manuscript (include quotes in quotation marks "like this" to indicate direct quotes from your manuscript), or elaborate on this item by providing additional information not in the ms, or briefly explain why the item is not applicable/relevant for your study

"Web-based software randomization and intervention delivery were completed using the Computerized Intervention Authoring System (CIAS), version 2.0., developed by Dr. Steven Ondersma."

5-ii) Describe the history/development process

Describe the history/development process of the application and previous formative evaluations (e.g., focus groups, usability testing), as these will have an impact on adoption/use rates and help with interpreting results.

	1	2	3	4	5	
subitem not at all important	○	○	○	○	○	essential

Does your paper address subitem 5-ii?

Copy and paste relevant sections from the manuscript (include quotes in quotation marks "like this" to indicate direct quotes from your manuscript), or elaborate on this item by providing additional information not in the ms, or briefly explain why the item is not applicable/relevant for your study

"The Health Check-Up for Expectant Moms (HCEM) is an innovative, theory-driven, technology-delivered brief intervention –– a single-session plus two booster sessions – designed to reduce alcohol/drug use and risk for STIs during pregnancy. In the HCEM pilot RCT, we found very high ratings of acceptability (between 6.3 and 6.8 on a 1-7 scale) in our sample of 50 pregnant women, and noted that the HCEM was associated with significantly larger reduction (54%) in any cannabis or alcohol use during pregnancy compared with participants in the control group (16%; P=.015)."

5-iii) Revisions and updating

Revisions and updating. Clearly mention the date and/or version number of the application/intervention (and comparator, if applicable) evaluated, or describe whether the intervention underwent major changes during the evaluation process, or whether the development and/or content was “frozen” during the trial. Describe dynamic components such as news feeds or changing content which may have an impact on the replicability of the intervention (for unexpected events see item 3b).

	1	2	3	4	5	
subitem not at all important	○	○	○	○	○	essential

Does your paper address subitem 5-iii?

Copy and paste relevant sections from the manuscript (include quotes in quotation marks "like this" to indicate direct quotes from your manuscript), or elaborate on this item by providing additional information not in the ms, or briefly explain why the item is not applicable/relevant for your study

Not applicable to our study as it's not a true, free-standing app that saw any version updates.

5-iv) Quality assurance methods

Provide information on quality assurance methods to ensure accuracy and quality of information provided [1], if applicable.

	1	2	3	4	5	
subitem not at all important	○	○	○	○	○	essential

Does your paper address subitem 5-iv?

Copy and paste relevant sections from the manuscript (include quotes in quotation marks "like this" to indicate direct quotes from your manuscript), or elaborate on this item by providing additional information not in the ms, or briefly explain why the item is not applicable/relevant for your study

"The Health Check-up for Expectant Moms (HCEM) is a 40-minute single session brief motivational interviewing (MI)-consistent intervention plus two booster sessions that is theory-driven and derived from empirical support. MI is a client-centered counseling style that facilitates internal motivation to change through alignment of behavior change with deeply held beliefs, values, and goals. MI has been found to be effective for STI/HIV risk reduction, and is consistent with Self Determination Theory as it utilizes evolving readiness and self-efficacy to change."

5-v) Ensure replicability by publishing the source code, and/or providing screenshots/screen-capture video, and/or providing flowcharts of the algorithms used

Ensure replicability by publishing the source code, and/or providing screenshots/screen-capture video, and/or providing flowcharts of the algorithms used. Replicability (i.e., other researchers should in principle be able to replicate the study) is a hallmark of scientific reporting.

	1	2	3	4	5	
subitem not at all important	○	○	○	○	○	essential

Does your paper address subitem 5-v?

Copy and paste relevant sections from the manuscript (include quotes in quotation marks "like this" to indicate direct quotes from your manuscript), or elaborate on this item by providing additional information not in the ms, or briefly explain why the item is not applicable/relevant for your study

Not applicable as our study website no longer exists. We created the intervention using CIAS 2.0, which is already indicated in the manuscript.
"Web-based software randomization and intervention delivery were completed using the Computerized Intervention Authoring System (CIAS), version 2.0., developed by Dr. Steven Ondersma."

5-vi) Digital preservation

Digital preservation: Provide the URL of the application, but as the intervention is likely to change or disappear over the course of the years; also make sure the intervention is archived (Internet Archive, webcitation.org, and/or publishing the source code or screenshots/videos alongside the article). As pages behind login screens cannot be archived, consider creating demo pages which are accessible without login.

	1	2	3	4	5	
subitem not at all important	○	○	○	○	○	essential

Does your paper address subitem 5-vi?

Copy and paste relevant sections from the manuscript (include quotes in quotation marks "like this" to indicate direct quotes from your manuscript), or elaborate on this item by providing additional information not in the ms, or briefly explain why the item is not applicable/relevant for your study

We could not do this as we did not own the rights to the CIAS software, and it has since been discontinued.

5-vii) Access

Access: Describe how participants accessed the application, in what setting/context, if they had to pay (or were paid) or not, whether they had to be a member of specific group. If known, describe how participants obtained “access to the platform and Internet” [1]. To ensure access for editors/reviewers/readers, consider to provide a “backdoor” login account or demo mode for reviewers/readers to explore the application (also important for archiving purposes, see vi).

	1	2	3	4	5	
subitem not at all important	○	○	○	○	○	essential

Does your paper address subitem 5-vii? *

Copy and paste relevant sections from the manuscript (include quotes in quotation marks "like this" to indicate direct quotes from your manuscript), or elaborate on this item by providing additional information not in the ms, or briefly explain why the item is not applicable/relevant for your study

"Participants completed the assessment and intervention or control sessions in the clinic on the study iPad (pre-COVID) or on a personal smartphone or computer/device through a web link to the study website (post-COVID). Booster sessions were emailed to participants to be completed independently using a web link to the study website both pre- and post-COVID-19."

5-viii) Mode of delivery, features/functionalities/components of the intervention and comparator, and the theoretical framework

Describe mode of delivery, features/functionalities/components of the intervention and comparator, and the theoretical framework [6] used to design them (instructional strategy [1], behaviour change techniques, persuasive features, etc., see e.g., [7, 8] for terminology). This includes an in-depth description of the content (including where it is coming from and who developed it) [1],” whether [and how] it is tailored to individual circumstances and allows users to track their progress and receive feedback” [6]. This also includes a description of communication delivery channels and – if computer-mediated communication is a component – whether communication was synchronous or asynchronous [6]. It also includes information on presentation strategies [1], including page design principles, average amount of text on pages, presence of hyperlinks to other resources, etc. [1].

	1	2	3	4	5	
subitem not at all important	○	○	○	○	○	essential

Does your paper address subitem 5-viii? *

Copy and paste relevant sections from the manuscript (include quotes in quotation marks "like this" to indicate direct quotes from your manuscript), or elaborate on this item by providing additional information not in the ms, or briefly explain why the item is not applicable/relevant for your study

"Participants completed the assessment and intervention or control sessions in the clinic on the study iPad (pre-COVID) or on a personal smartphone or computer/device through a web link to the study website (post-COVID). Booster sessions were emailed to participants to be completed independently using a web link to the study website both pre- and post-COVID-19. "

AND

"The Health Check-up for Expectant Moms (HCEM) is a 40-minute single session brief motivational interviewing (MI)-consistent intervention plus two booster sessions that is theory-driven and derived from empirical support. MI is a client-centered counseling style that facilitates internal motivation to change through alignment of behavior change with deeply held beliefs, values, and goals. MI has been found to be effective for STI/HIV risk reduction, and is consistent with Self Determination Theory as it utilizes evolving readiness and self-efficacy to change. The focus of the intervention session is on reducing the key behavioral outcomes of unprotected sex and alcohol/drug use during pregnancy. The session provides a tutorial guided by a narrator which interacts in a collaborative, MI-consistent style. HCEM includes short video testimonials of women who had an STI, how it affected them during pregnancy, and how they sought support...

Within one month of completing the intervention session, participants in the intervention group complete two, 15-20-minute booster sessions, also delivered remotely. For participants in the HCEM condition, the booster sessions review their own personalized plan and identify any challenges or barriers to increasing healthy behaviors (i.e., increasing condom use, improving supports). Booster sessions explicitly target the prevention or reduction of STI risk behaviors including alcohol, drug use during the postpartum period, when prevalence of these behaviors increases, and address health behaviors associated with postpartum substance use (e.g., risks of substance use while breastfeeding).

Our control condition is a well-validated, attention, time, and information-matched control that was successfully included in our previous studies, including in our prior HCEM trial. The content consists of a series of questions regarding television show preferences and viewing a brief series of videos of popular entertainers/shows, with subsequent requests for ratings of subjective preference. Participants randomized to the control condition also receive facts about alcohol/drug use and risky sex during pregnancy through informational brochures from National Institutes of Health (NIH) and the CDC that also provided face validity. Participants in the control condition receive booster content that is similar to the baseline content."

5-ix) Describe use parameters

Describe use parameters (e.g., intended “doses” and optimal timing for use). Clarify what instructions or recommendations were given to the user, e.g., regarding timing, frequency, heaviness of use, if any, or was the intervention used ad libitum.

	1	2	3	4	5	
subitem not at all important	○	○	○	○	○	essential

Does your paper address subitem 5-ix?

Copy and paste relevant sections from the manuscript (include quotes in quotation marks "like this" to indicate direct quotes from your manuscript), or elaborate on this item by providing additional information not in the ms, or briefly explain why the item is not applicable/relevant for your study

"..an innovative, theory-driven, technology-delivered and fully automated brief intervention (single session plus two booster sessions).."

5-x) Clarify the level of human involvement

Clarify the level of human involvement (care providers or health professionals, also technical assistance) in the e-intervention or as co-intervention (detail number and expertise of professionals involved, if any, as well as “type of assistance offered, the timing and frequency of the support, how it is initiated, and the medium by which the assistance is delivered”. It may be necessary to distinguish between the level of human involvement required for the trial, and the level of human involvement required for a routine application outside of a RCT setting (discuss under item 21 – generalizability).

	1	2	3	4	5	
subitem not at all important	○	○	○	○	○	essential

Does your paper address subitem 5-x?

Copy and paste relevant sections from the manuscript (include quotes in quotation marks "like this" to indicate direct quotes from your manuscript), or elaborate on this item by providing additional information not in the ms, or briefly explain why the item is not applicable/relevant for your study

"Initial participant recruitment took place in-person at family medicine and obstetrician/gynecology clinics between April 2019 and March 2020 (n = 81). Due to the COVID-19 pandemic, new recruitment stopped until August 2020, to allow time to make the necessary protocol and software changes for complete remote delivery (see previous paper for more detail). Remote recruitment began in August 2020 and ended in September 2023 (n = 95). Remote recruitment included calling/texting/emailing existing clinic patients and social media advertising (Facebook® and Instagram®) throughout the state of Michigan."

AND

"Participants completed the assessment and intervention or control sessions in the clinic on the study iPad (pre-COVID) or on a personal smartphone or computer/device through a web link to the study website (post-COVID). Booster sessions were emailed to participants to be completed independently using a web link to the study website both pre- and post-COVID-19."

AND

"We collected biological hair samples (Psychemedics, Inc.) and/or urine samples in person at the baseline and 6-month follow-up assessment to corroborate self-report of drug use (a secondary outcome). The hair samples were obtained by a trained study RA, collected from cosmetically undetectable areas on the scalp and sent off-site for analysis. All biological sample collection was suspended during the COVID-19 pandemic and therefore this data was incomplete for all participants."

AND

"Participants completed the assessment and intervention or control sessions in the clinic on the study iPad (pre-COVID) or on a personal smartphone or computer/device through a web link to the study website (post-COVID). Booster sessions were emailed to participants to be completed independently using a web link to the study website both pre- and post-COVID-19. Reminders by phone/text/email to complete booster sessions were sent as needed until the 2-month follow-up visit, after which booster sessions were no longer available to complete. Whether in-person or remote, a research study team member was available (during regular business hours) to help participants troubleshoot any technical problems. All participant contact (call/text/email) was conducted using university-secured and encrypted websites to ensure participant confidentiality and safety."

AND

"2) Timeline Follow-Back (TLFB) calendar interview was conducted by the research assistant either in-person (pre-COVID-19) or remotely by phone."

5-xi) Report any prompts/reminders used

Report any prompts/reminders used: Clarify if there were prompts (letters, emails, phone calls, SMS) to use the application, what triggered them, frequency etc. It may be necessary to distinguish between the level of prompts/reminders required for the trial, and the level of prompts/reminders for a routine application outside of a RCT setting (discuss under item 21 – generalizability).

	1	2	3	4	5	
subitem not at all important	○	○	○	○	○	essential

Does your paper address subitem 5-xi? *

Copy and paste relevant sections from the manuscript (include quotes in quotation marks "like this" to indicate direct quotes from your manuscript), or elaborate on this item by providing additional information not in the ms, or briefly explain why the item is not applicable/relevant for your study

"Reminders by phone/text/email to complete booster sessions were sent as needed until the 2-month follow-up visit, after which booster sessions were no longer available to complete."

5-xii) Describe any co-interventions (incl. training/support)

Describe any co-interventions (incl. training/support): Clearly state any interventions that are provided in addition to the targeted eHealth intervention, as ehealth intervention may not be designed as stand-alone intervention. This includes training sessions and support [1]. It may be necessary to distinguish between the level of training required for the trial, and the level of training for a routine application outside of a RCT setting (discuss under item 21 – generalizability.

	1	2	3	4	5	
subitem not at all important	○	○	○	○	○	essential

Does your paper address subitem 5-xii? *

Copy and paste relevant sections from the manuscript (include quotes in quotation marks "like this" to indicate direct quotes from your manuscript), or elaborate on this item by providing additional information not in the ms, or briefly explain why the item is not applicable/relevant for your study

Not applicable to this study.

6a) Completely defined pre-specified primary and secondary outcome measures, including how and when they were assessed

Does your paper address CONSORT subitem 6a? *

Copy and paste relevant sections from the manuscript (include quotes in quotation marks "like this" to indicate direct quotes from your manuscript), or elaborate on this item by providing additional information not in the ms, or briefly explain why the item is not applicable/relevant for your study

"Building upon this pilot work, we report the results of our randomized controlled trial testing whether the HCEM reduced condomless sex/STI risk, alcohol/drug use, more than a control condition in a larger, more diverse sample of pregnant women seeking prenatal care."

AND

"Study Measures for Primary Outcomes
Participants completed an approximately 25-minute assessment session before starting the intervention or control session. The assessment battery was designed to minimize assessment with the time, attention, and information-matched control group to account for concerns regarding the motivational properties of assessment. All of the self-report measures have good psychometric properties:
1) Demographic information included age, race, ethnicity, marital/partner status, parity, employment, socioeconomic status. Web-based collection was used both pre- and post-COVID-19 for all demographic information.
2) Timeline Follow-Back (TLFB) calendar interview was conducted by the research assistant either in-person (pre-COVID-19) or remotely by phone. Data for the TLFB only was entered into a database only accessible by the research team, which differs from other assessment measures that were collected through direct input from the participant either on the study iPad or via web link to the study website. The TLFB is a calendar assisted structured interview that provides a way to cue memory so that accurate recall is enhanced for event-level data; it has been used to assess sexual risk-taking. It assessed sexual health behaviors placing women at risk for STIs at baseline and follow up assessments. A partner-by-partner assessment of sexual behaviors and condom use was made for each of the participant's sexual partners and partner type (casual, main). Condomless sex assessed in the 90 days prior to the baseline, as well as for each of the subsequent assessments (at 2- and 6-months post-baseline, antenatally). The 90-day time period was selected because of research supporting reliable reporting of sexual events and number of partners over this time period. The TLFB was also used to measure drinks per drinking day, percent days abstinent, and heavy episodic drinking episodes; it was also used to measure other drug use, including cannabis use."

6a-i) Online questionnaires: describe if they were validated for online use and apply CHERRIES items to describe how the questionnaires were designed/deployed

If outcomes were obtained through online questionnaires, describe if they were validated for online use and apply CHERRIES items to describe how the questionnaires were designed/deployed [9].

	1	2	3	4	5	
subitem not at all important	○	○	○	○	○	essential

Does your paper address subitem 6a-i?

Copy and paste relevant sections from manuscript text

Not applicable to this study.

6a-ii) Describe whether and how “use” (including intensity of use/dosage) was defined/measured/monitored

Describe whether and how “use” (including intensity of use/dosage) was defined/measured/monitored (logins, logfile analysis, etc.). Use/adoption metrics are important process outcomes that should be reported in any ehealth trial.

	1	2	3	4	5	
subitem not at all important	○	○	○	○	○	essential

Does your paper address subitem 6a-ii?

Copy and paste relevant sections from manuscript text

Not applicable to this study.

6a-iii) Describe whether, how, and when qualitative feedback from participants was obtained

Describe whether, how, and when qualitative feedback from participants was obtained (e.g., through emails, feedback forms, interviews, focus groups).

	1	2	3	4	5	
subitem not at all important	○	○	○	○	○	essential

Does your paper address subitem 6a-iii?

Copy and paste relevant sections from manuscript text

This isn't applicable to the current paper as qualitative feedback wasn't used for the creation of HCEM, just positive feedback from the pilot, and none of the primary outcomes were qualitative.

6b) Any changes to trial outcomes after the trial commenced, with reasons

Does your paper address CONSORT subitem 6b? *

Copy and paste relevant sections from the manuscript (include quotes in quotation marks "like this" to indicate direct quotes from your manuscript), or elaborate on this item by providing additional information not in the ms, or briefly explain why the item is not applicable/relevant for your study

No, there were no changes to the trial outcomes.

7a) How sample size was determined

NPT: When applicable, details of whether and how the clustering by care provides or centers was addressed

7a-i) Describe whether and how expected attrition was taken into account when calculating the sample size

Describe whether and how expected attrition was taken into account when calculating the sample size.

	1	2	3	4	5	
subitem not at all important	○	○	○	○	○	essential

Does your paper address subitem 7a-i?

Copy and paste relevant sections from manuscript title (include quotes in quotation marks "like this" to indicate direct quotes from your manuscript), or elaborate on this item by providing additional information not in the ms, or briefly explain why the item is not applicable/relevant for your study

"We estimated power based on our R21 study and calculated it using extensive simulation..."

7b) When applicable, explanation of any interim analyses and stopping guidelines

Does your paper address CONSORT subitem 7b? *

Copy and paste relevant sections from the manuscript (include quotes in quotation marks "like this" to indicate direct quotes from your manuscript), or elaborate on this item by providing additional information not in the ms, or briefly explain why the item is not applicable/relevant for your study

Not applicable to the current study.

8a) Method used to generate the random allocation sequence

NPT: When applicable, how care providers were allocated to each trial group

Does your paper address CONSORT subitem 8a? *

Copy and paste relevant sections from the manuscript (include quotes in quotation marks "like this" to indicate direct quotes from your manuscript), or elaborate on this item by providing additional information not in the ms, or briefly explain why the item is not applicable/relevant for your study

"Using simple randomization, we computer randomized 176 pregnant women who met study inclusion criteria into the HCEM intervention (n = 88) or an attention, time, and information-matched control (n=88) condition."

8b) Type of randomisation; details of any restriction (such as blocking and block size)

Does your paper address CONSORT subitem 8b? *

Copy and paste relevant sections from the manuscript (include quotes in quotation marks "like this" to indicate direct quotes from your manuscript), or elaborate on this item by providing additional information not in the ms, or briefly explain why the item is not applicable/relevant for your study

"Using simple randomization, we computer randomized 176 pregnant women who met study inclusion criteria into the HCEM intervention (n = 88) or an attention, time, and information-matched control (n=88) condition."

9) Mechanism used to implement the random allocation sequence (such as sequentially numbered containers), describing any steps taken to conceal the sequence until interventions were assigned

Does your paper address CONSORT subitem 9? *

Copy and paste relevant sections from the manuscript (include quotes in quotation marks "like this" to indicate direct quotes from your manuscript), or elaborate on this item by providing additional information not in the ms, or briefly explain why the item is not applicable/relevant for your study

"Using simple randomization, we computer randomized 176 pregnant women who met study inclusion criteria into the HCEM intervention (n = 88) or an attention, time, and information-matched control (n=88) condition."

10) Who generated the random allocation sequence, who enrolled participants, and who assigned participants to interventions

Does your paper address CONSORT subitem 10? *

Copy and paste relevant sections from the manuscript (include quotes in quotation marks "like this" to indicate direct quotes from your manuscript), or elaborate on this item by providing additional information not in the ms, or briefly explain why the item is not applicable/relevant for your study

"Using simple randomization, we computer randomized 176 pregnant women who met study inclusion criteria into the HCEM intervention (n = 88) or an attention, time, and information-matched control (n=88) condition."

AND

"All participants went through a detailed informed consent process with a research staff member before being enrolled in the study."

11a) If done, who was blinded after assignment to interventions (for example, participants, care providers, those assessing outcomes) and how

NPT: Whether or not administering co-interventions were blinded to group assignment

11a-i) Specify who was blinded, and who wasn't

Specify who was blinded, and who wasn't. Usually, in web-based trials it is not possible to blind the participants [1, 3] (this should be clearly acknowledged), but it may be possible to blind outcome assessors, those doing data analysis or those administering co-interventions (if any).

	1	2	3	4	5	
subitem not at all important	○	○	○	○	○	essential

Does your paper address subitem 11a-i? *

Copy and paste relevant sections from the manuscript (include quotes in quotation marks "like this" to indicate direct quotes from your manuscript), or elaborate on this item by providing additional information not in the ms, or briefly explain why the item is not applicable/relevant for your study

"Study staff members were blinded to the arm allocations of the participants they interviewed at the 2- and 6-month follow-up visits. This meant that the staff member who conducted the baseline session for a participant could not conduct any follow-up visits for that same participant. Participants were not told which arm they were randomized to, however, it is possible for them to have inferred it after reading the informed consent document, as it detailed the two study conditions."

11a-ii) Discuss e.g., whether participants knew which intervention was the "intervention of interest" and which one was the "comparator"

Informed consent procedures (4a-ii) can create biases and certain expectations - discuss e.g., whether participants knew which intervention was the "intervention of interest" and which one was the "comparator".

	1	2	3	4	5	
subitem not at all important	○	○	○	○	○	essential

Does your paper address subitem 11a-ii?

Copy and paste relevant sections from the manuscript (include quotes in quotation marks "like this" to indicate direct quotes from your manuscript), or elaborate on this item by providing additional information not in the ms, or briefly explain why the item is not applicable/relevant for your study

"Participants were not told which arm they were randomized to, however, it is possible for them to have inferred it after reading the informed consent document, as it detailed the two study conditions."

11b) If relevant, description of the similarity of interventions

(this item is usually not relevant for ehealth trials as it refers to similarity of a placebo or sham intervention to a active medication/intervention)

Does your paper address CONSORT subitem 11b? *

Copy and paste relevant sections from the manuscript (include quotes in quotation marks "like this" to indicate direct quotes from your manuscript), or elaborate on this item by providing additional information not in the ms, or briefly explain why the item is not applicable/relevant for your study

Your answer

12a) Statistical methods used to compare groups for primary and secondary outcomes

NPT: When applicable, details of whether and how the clustering by care providers or centers was addressed

Does your paper address CONSORT subitem 12a? *

Copy and paste relevant sections from the manuscript (include quotes in quotation marks "like this" to indicate direct quotes from your manuscript), or elaborate on this item by providing additional information not in the ms, or briefly explain why the item is not applicable/relevant for your study

"We summarized key study variables through descriptive statistics. Participant characteristics and outcomes at three timepoints during pregnancy were compared between control and intervention groups by means of two sample t-tests (continuous), Wilcoxon rank sum tests, chi-square tests, and Fisher's exact tests, as appropriate. The primary behaviors of interest were alcohol or drug use, (primarily cannabis use), and unprotected sexual occasions, as assessed by the TLFB."

12a-i) Imputation techniques to deal with attrition / missing values

Imputation techniques to deal with attrition / missing values: Not all participants will use the intervention/comparator as intended and attrition is typically high in ehealth trials. Specify how participants who did not use the application or dropped out from the trial were treated in the statistical analysis (a complete case analysis is strongly discouraged, and simple imputation techniques such as LOCF may also be problematic [4]).

	1	2	3	4	5	
subitem not at all important	○	○	○	○	○	essential

Does your paper address subitem 12a-i? *

Copy and paste relevant sections from the manuscript (include quotes in quotation marks "like this" to indicate direct quotes from your manuscript), or elaborate on this item by providing additional information not in the ms, or briefly explain why the item is not applicable/relevant for your study

This is not applicable to our study.

12b) Methods for additional analyses, such as subgroup analyses and adjusted analyses

Does your paper address CONSORT subitem 12b? *

Copy and paste relevant sections from the manuscript (include quotes in quotation marks "like this" to indicate direct quotes from your manuscript), or elaborate on this item by providing additional information not in the ms, or briefly explain why the item is not applicable/relevant for your study

This is not applicable to our study.

X26) REB/IRB Approval and Ethical Considerations [recommended as subheading under "Methods"] (not a CONSORT item)

X26-i) Comment on ethics committee approval

	1	2	3	4	5	
subitem not at all important	○	○	○	○	○	essential

Does your paper address subitem X26-i?

Copy and paste relevant sections from the manuscript (include quotes in quotation marks "like this" to indicate direct quotes from your manuscript), or elaborate on this item by providing additional information not in the ms, or briefly explain why the item is not applicable/relevant for your study

"The study was approved on April 23, 2018 (HUM00143896) by the Institutional Review Board of the University of Michigan Medical School in Ann Arbor, MI and is registered on ClinicalTrials.gov. All participants went through a detailed informed consent process with a research staff member before being enrolled in the study. Informed consent was obtained face-to-face with signatures pre-COVID-19, and over the phone with verbal consent post-COVID-19."

x26-ii) Outline informed consent procedures

Outline informed consent procedures e.g., if consent was obtained offline or online (how? Checkbox, etc.?), and what information was provided (see 4a-ii). See [6] for some items to be included in informed consent documents.

	1	2	3	4	5	
subitem not at all important	○	○	○	○	○	essential

Does your paper address subitem X26-ii?

Copy and paste relevant sections from the manuscript (include quotes in quotation marks "like this" to indicate direct quotes from your manuscript), or elaborate on this item by providing additional information not in the ms, or briefly explain why the item is not applicable/relevant for your study

"All participants went through a detailed informed consent process with a research staff member before being enrolled in the study. Informed consent was obtained face-to-face with signatures pre-COVID-19, and over the phone with verbal consent post-COVID-19."

X26-iii) Safety and security procedures

Safety and security procedures, incl. privacy considerations, and any steps taken to reduce the likelihood or detection of harm (e.g., education and training, availability of a hotline)

	1	2	3	4	5	
subitem not at all important	○	○	○	○	○	essential

Does your paper address subitem X26-iii?

Copy and paste relevant sections from the manuscript (include quotes in quotation marks "like this" to indicate direct quotes from your manuscript), or elaborate on this item by providing additional information not in the ms, or briefly explain why the item is not applicable/relevant for your study

"All participant contact (call/text/email) was conducted using university-secured and encrypted websites to ensure participant confidentiality and safety."

RESULTS

13a) For each group, the numbers of participants who were randomly assigned, received intended treatment, and were analysed for the primary outcome

NPT: The number of care providers or centers performing the intervention in each group and the number of patients treated by each care provider in each center

Does your paper address CONSORT subitem 13a? *

Copy and paste relevant sections from the manuscript (include quotes in quotation marks "like this" to indicate direct quotes from your manuscript), or elaborate on this item by providing additional information not in the ms, or briefly explain why the item is not applicable/relevant for your study

"Overall, 180 women were enrolled for the randomized trial (8% of the women who were assessed for eligibility), 176 of them (97.7%) were randomized, and 174 completed both the baseline assessment and the intervention session."

13b) For each group, losses and exclusions after randomisation, together with reasons

Does your paper address CONSORT subitem 13b? (NOTE: Preferably, this is shown in a CONSORT flow diagram) *

Copy and paste relevant sections from the manuscript (include quotes in quotation marks "like this" to indicate direct quotes from your manuscript), or elaborate on this item by providing additional information not in the ms, or briefly explain why the item is not applicable/relevant for your study

"Of the 88 women randomized to the intervention condition, 72 (82%) completed both booster sessions..."

13b-i) Attrition diagram

Strongly recommended: An attrition diagram (e.g., proportion of participants still logging in or using the intervention/comparator in each group plotted over time, similar to a survival curve) or other figures or tables demonstrating usage/dose/engagement.

	1	2	3	4	5	
subitem not at all important	○	○	○	○	○	essential

Does your paper address subitem 13b-i?

Copy and paste relevant sections from the manuscript or cite the figure number if applicable (include quotes in quotation marks "like this" to indicate direct quotes from your manuscript), or elaborate on this item by providing additional information not in the ms, or briefly explain why the item is not applicable/relevant for your study

We will include a CONSORT diagram.

14a) Dates defining the periods of recruitment and follow-up

Does your paper address CONSORT subitem 14a? *

Copy and paste relevant sections from the manuscript (include quotes in quotation marks "like this" to indicate direct quotes from your manuscript), or elaborate on this item by providing additional information not in the ms, or briefly explain why the item is not applicable/relevant for your study

"Initial participant recruitment took place in-person at family medicine and obstetrician/gynecology clinics between April 2019 and March 2020 (n = 81)..."

14a-i) Indicate if critical "secular events" fell into the study period

Indicate if critical "secular events" fell into the study period, e.g., significant changes in Internet resources available or "changes in computer hardware or Internet delivery resources"

	1	2	3	4	5	
subitem not at all important	○	○	○	○	○	essential

Does your paper address subitem 14a-i?

Copy and paste relevant sections from the manuscript (include quotes in quotation marks "like this" to indicate direct quotes from your manuscript), or elaborate on this item by providing additional information not in the ms, or briefly explain why the item is not applicable/relevant for your study

"Due to the COVID-19 pandemic, new recruitment stopped until August 2020.."

14b) Why the trial ended or was stopped (early)

Does your paper address CONSORT subitem 14b? *

Copy and paste relevant sections from the manuscript (include quotes in quotation marks "like this" to indicate direct quotes from your manuscript), or elaborate on this item by providing additional information not in the ms, or briefly explain why the item is not applicable/relevant for your study

Your answer

15) A table showing baseline demographic and clinical characteristics for each group

NPT: When applicable, a description of care providers (case volume, qualification, expertise, etc.) and centers (volume) in each group

Does your paper address CONSORT subitem 15? *

Copy and paste relevant sections from the manuscript (include quotes in quotation marks "like this" to indicate direct quotes from your manuscript), or elaborate on this item by providing additional information not in the ms, or briefly explain why the item is not applicable/relevant for your study

We include this as Table 1.

15-i) Report demographics associated with digital divide issues

In ehealth trials it is particularly important to report demographics associated with digital divide issues, such as age, education, gender, social-economic status, computer/Internet/ehealth literacy of the participants, if known.

	1	2	3	4	5	
subitem not at all important	○	○	○	○	○	essential

Does your paper address subitem 15-i? *

Copy and paste relevant sections from the manuscript (include quotes in quotation marks "like this" to indicate direct quotes from your manuscript), or elaborate on this item by providing additional information not in the ms, or briefly explain why the item is not applicable/relevant for your study

This was not assessed in this study.

16) For each group, number of participants (denominator) included in each analysis and whether the analysis was by original assigned groups

16-i) Report multiple “denominators” and provide definitions

Report multiple “denominators” and provide definitions: Report N’s (and effect sizes) “across a range of study participation [and use] thresholds” [1], e.g., N exposed, N consented, N used more than x times, N used more than y weeks, N participants “used” the intervention/comparator at specific pre-defined time points of interest (in absolute and relative numbers per group). Always clearly define “use” of the intervention.

	1	2	3	4	5	
subitem not at all important	○	○	○	○	○	essential

Does your paper address subitem 16-i? *

Copy and paste relevant sections from the manuscript (include quotes in quotation marks "like this" to indicate direct quotes from your manuscript), or elaborate on this item by providing additional information not in the ms, or briefly explain why the item is not applicable/relevant for your study

"..we computer randomized 176 pregnant women who met study inclusion criteria into the HCEM intervention (n = 88) or an attention, time, and information-matched control (n=88) condition."

16-ii) Primary analysis should be intent-to-treat

Primary analysis should be intent-to-treat, secondary analyses could include comparing only “users”, with the appropriate caveats that this is no longer a randomized sample (see 18-i).

	1	2	3	4	5	
subitem not at all important	○	○	○	○	○	essential

Does your paper address subitem 16-ii?

Copy and paste relevant sections from the manuscript (include quotes in quotation marks "like this" to indicate direct quotes from your manuscript), or elaborate on this item by providing additional information not in the ms, or briefly explain why the item is not applicable/relevant for your study

"Analyses were conducted according to the intent-to-treat principle."

17a) For each primary and secondary outcome, results for each group, and the estimated effect size and its precision (such as 95% confidence interval)

Does your paper address CONSORT subitem 17a? *

Copy and paste relevant sections from the manuscript (include quotes in quotation marks "like this" to indicate direct quotes from your manuscript), or elaborate on this item by providing additional information not in the ms, or briefly explain why the item is not applicable/relevant for your study

"In the adjusted model, compared with baseline, the HCEM group had an 93% reduction at 2 months (AOR = 0.07, 95% CI: 0.03–0.17, P<.001) and a 95% reduction at 6 months (AOR = 0.05, 95% CI: 0.02–0.12, P<.001)."

17a-i) Presentation of process outcomes such as metrics of use and intensity of use

In addition to primary/secondary (clinical) outcomes, the presentation of process outcomes such as metrics of use and intensity of use (dose, exposure) and their operational definitions is critical. This does not only refer to metrics of attrition (13-b) (often a binary variable), but also to more continuous exposure metrics such as “average session length”. These must be accompanied by a technical description how a metric like a “session” is defined (e.g., timeout after idle time) [1] (report under item 6a).

	1	2	3	4	5	
subitem not at all important	○	○	○	○	○	essential

Does your paper address subitem 17a-i?

Copy and paste relevant sections from the manuscript (include quotes in quotation marks "like this" to indicate direct quotes from your manuscript), or elaborate on this item by providing additional information not in the ms, or briefly explain why the item is not applicable/relevant for your study

Not applicable to current study.

17b) For binary outcomes, presentation of both absolute and relative effect sizes is recommended

Does your paper address CONSORT subitem 17b? *

Copy and paste relevant sections from the manuscript (include quotes in quotation marks "like this" to indicate direct quotes from your manuscript), or elaborate on this item by providing additional information not in the ms, or briefly explain why the item is not applicable/relevant for your study

This study used time-by-group interaction.

18) Results of any other analyses performed, including subgroup analyses and adjusted analyses, distinguishing pre-specified from exploratory

Does your paper address CONSORT subitem 18? *

Copy and paste relevant sections from the manuscript (include quotes in quotation marks "like this" to indicate direct quotes from your manuscript), or elaborate on this item by providing additional information not in the ms, or briefly explain why the item is not applicable/relevant for your study

Not applicable to current study.

18-i) Subgroup analysis of comparing only users

A subgroup analysis of comparing only users is not uncommon in ehealth trials, but if done, it must be stressed that this is a self-selected sample and no longer an unbiased sample from a randomized trial (see 16-iii).

	1	2	3	4	5	
subitem not at all important	○	○	○	○	○	essential

Does your paper address subitem 18-i?

Copy and paste relevant sections from the manuscript (include quotes in quotation marks "like this" to indicate direct quotes from your manuscript), or elaborate on this item by providing additional information not in the ms, or briefly explain why the item is not applicable/relevant for your study

Your answer

19) All important harms or unintended effects in each group

(for specific guidance see CONSORT for harms)

Does your paper address CONSORT subitem 19? *

Copy and paste relevant sections from the manuscript (include quotes in quotation marks "like this" to indicate direct quotes from your manuscript), or elaborate on this item by providing additional information not in the ms, or briefly explain why the item is not applicable/relevant for your study

Not applicable to current study.

19-i) Include privacy breaches, technical problems

Include privacy breaches, technical problems. This does not only include physical "harm" to participants, but also incidents such as perceived or real privacy breaches [1], technical problems, and other unexpected/unintended incidents. "Unintended effects" also includes unintended positive effects [2].

	1	2	3	4	5	
subitem not at all important	○	○	○	○	○	essential

Does your paper address subitem 19-i?

Copy and paste relevant sections from the manuscript (include quotes in quotation marks "like this" to indicate direct quotes from your manuscript), or elaborate on this item by providing additional information not in the ms, or briefly explain why the item is not applicable/relevant for your study

Not applicable to current study.

19-ii) Include qualitative feedback from participants or observations from staff/researchers

Include qualitative feedback from participants or observations from staff/researchers, if available, on strengths and shortcomings of the application, especially if they point to unintended/unexpected effects or uses. This includes (if available) reasons for why people did or did not use the application as intended by the developers.

	1	2	3	4	5	
subitem not at all important	○	○	○	○	○	essential

Does your paper address subitem 19-ii?

Copy and paste relevant sections from the manuscript (include quotes in quotation marks "like this" to indicate direct quotes from your manuscript), or elaborate on this item by providing additional information not in the ms, or briefly explain why the item is not applicable/relevant for your study

Not applicable to current study.

DISCUSSION

22) Interpretation consistent with results, balancing benefits and harms, and considering other relevant evidence

NPT: In addition, take into account the choice of the comparator, lack of or partial blinding, and unequal expertise of care providers or centers in each group

22-i) Restate study questions and summarize the answers suggested by the data, starting with primary outcomes and process outcomes (use)

Restate study questions and summarize the answers suggested by the data, starting with primary outcomes and process outcomes (use).

	1	2	3	4	5	
subitem not at all important	○	○	○	○	○	essential

Does your paper address subitem 22-i? *

Copy and paste relevant sections from the manuscript (include quotes in quotation marks "like this" to indicate direct quotes from your manuscript), or elaborate on this item by providing additional information not in the ms, or briefly explain why the item is not applicable/relevant for your study

"The objective of this study was to test whether the web-based HCEM intervention reduced condomless sex/STI risk, alcohol, or drug use, more than an attention, time, and information-matched control condition in pregnant women seeking prenatal care."

22-ii) Highlight unanswered new questions, suggest future research

Highlight unanswered new questions, suggest future research.

	1	2	3	4	5	
subitem not at all important	○	○	○	○	○	essential

Does your paper address subitem 22-ii?

Copy and paste relevant sections from the manuscript (include quotes in quotation marks "like this" to indicate direct quotes from your manuscript), or elaborate on this item by providing additional information not in the ms, or briefly explain why the item is not applicable/relevant for your study

"While both conditions were associated with reductions in alcohol/drug use that for the majority of participants began in the month prior to study enrollment, an intervention effect may have been impossible to observe given substantial self change. Additional factors include our small sample and the unique impacts of the COVID-19 pandemic on our study. Therefore, future studies should focus on women at higher risk of continued alcohol use after knowing they are pregnant."

20) Trial limitations, addressing sources of potential bias, imprecision, and, if relevant, multiplicity of analyses

20-i) Typical limitations in ehealth trials

Typical limitations in ehealth trials: Participants in ehealth trials are rarely blinded. Ehealth trials often look at a multiplicity of outcomes, increasing risk for a Type I error. Discuss biases due to non-use of the intervention/usability issues, biases through informed consent procedures, unexpected events.

	1	2	3	4	5	
subitem not at all important	O	O	O	O	O	essential

Does your paper address subitem 20-i? *

Copy and paste relevant sections from the manuscript (include quotes in quotation marks "like this" to indicate direct quotes from your manuscript), or elaborate on this item by providing additional information not in the ms, or briefly explain why the item is not applicable/relevant for your study

"The limitations of our study include a relatively small sample size, a highly educated sample, a limited geographical sample in one midwestern state and inability to generalize our results to other pregnant individuals."

21) Generalisability (external validity, applicability) of the trial findings
NPT: External validity of the trial findings according to the intervention, comparators, patients, and care providers or centers involved in the trial

21-i) Generalizability to other populations
Generalizability to other populations: In particular, discuss generalizability to a general Internet population, outside of a RCT setting, and general patient population, including applicability of the study results for other organizations

	1	2	3	4	5	
subitem not at all important	○	○	○	○	○	essential

Does your paper address subitem 21-i?
Copy and paste relevant sections from the manuscript (include quotes in quotation marks "like this" to indicate direct quotes from your manuscript), or elaborate on this item by providing additional information not in the ms, or briefly explain why the item is not applicable/relevant for your study

"The limitations of our study include a relatively small sample size, a highly educated sample, a limited geographical sample in one midwestern state and inability to generalize our results to other pregnant individuals."

21-ii) Discuss if there were elements in the RCT that would be different in a routine application setting
Discuss if there were elements in the RCT that would be different in a routine application setting (e.g., prompts/reminders, more human involvement, training sessions or other co-interventions) and what impact the omission of these elements could have on use, adoption, or outcomes if the intervention is applied outside of a RCT setting.

	1	2	3	4	5	
subitem not at all important	○	○	○	○	○	essential

Does your paper address subitem 21-ii?

Copy and paste relevant sections from the manuscript (include quotes in quotation marks "like this" to indicate direct quotes from your manuscript), or elaborate on this item by providing additional information not in the ms, or briefly explain why the item is not applicable/relevant for your study

This isn't in the current manuscript, submitted a separate paper about this topic.

OTHER INFORMATION

23) Registration number and name of trial registry

Does your paper address CONSORT subitem 23? *

Copy and paste relevant sections from the manuscript (include quotes in quotation marks "like this" to indicate direct quotes from your manuscript), or elaborate on this item by providing additional information not in the ms, or briefly explain why the item is not applicable/relevant for your study

"The study was approved on April 23, 2018 (HUM00143896) by the Institutional Review Board of the University of Michigan Medical School in Ann Arbor, MI and is registered on ClinicalTrials.gov (NCT03826342)."

24) Where the full trial protocol can be accessed, if available

Does your paper address CONSORT subitem 24? *

Cite a Multimedia Appendix, other reference, or copy and paste relevant sections from the manuscript (include quotes in quotation marks "like this" to indicate direct quotes from your manuscript), or elaborate on this item by providing additional information not in the ms, or briefly explain why the item is not applicable/relevant for your study

We can append to ms if needed.

25) Sources of funding and other support (such as supply of drugs), role of funders

Does your paper address CONSORT subitem 25? *

Copy and paste relevant sections from the manuscript (include quotes in quotation marks "like this" to indicate direct quotes from your manuscript), or elaborate on this item by providing additional information not in the ms, or briefly explain why the item is not applicable/relevant for your study

"This study was funded by the Eunice Kennedy Shriver National Institute of Child Health and Human Development (R01HD093611; PI: Tzilos Wernette)."

X27) Conflicts of Interest (not a CONSORT item)

X27-i) State the relation of the study team towards the system being evaluated

In addition to the usual declaration of interests (financial or otherwise), also state the relation of the study team towards the system being evaluated, i.e., state if the authors/evaluators are distinct from or identical with the developers/sponsors of the intervention.

	1	2	3	4	5	
subitem not at all important	○	○	○	○	○	essential

Does your paper address subitem X27-i?

Copy and paste relevant sections from the manuscript (include quotes in quotation marks "like this" to indicate direct quotes from your manuscript), or elaborate on this item by providing additional information not in the ms, or briefly explain why the item is not applicable/relevant for your study

"No conflicts of interest."

About the CONSORT EHEALTH checklist

As a result of using this checklist, did you make changes in your manuscript? *

- ( ) yes, major changes
- (x) yes, minor changes
- ( ) no

What were the most important changes you made as a result of using this checklist?

Your answer

How much time did you spend on going through the checklist INCLUDING making changes in your manuscript *

We estimate at least 10 hours.

As a result of using this checklist, do you think your manuscript has improved? *

- (x) yes
- ( ) no
- ( ) Other:

Would you like to become involved in the CONSORT EHEALTH group?

This would involve for example becoming involved in participating in a workshop and writing an "Explanation and Elaboration" document

- ( ) yes
- (x) no
- ( ) Other:

Clear selection

Any other comments or questions on CONSORT EHEALTH

Your answer

STOP - Save this form as PDF before you click submit

To generate a record that you filled in this form, we recommend to generate a PDF of this page (on a Mac, simply select "print" and then select "print as PDF") before you submit it.

When you submit your (revised) paper to JMIR, please upload the PDF as supplementary file.

Don't worry if some text in the textboxes is cut off, as we still have the complete information in our database. Thank you!

Final step: Click submit !

Click submit so we have your answers in our database!

Submit

Clear form

Never submit passwords through Google Forms.

This content is neither created nor endorsed by Google. - Contact form owner - Terms of Service - Privacy Policy

Does this form look suspicious? Report

Google Forms
